# Agentic AI for smart and sustainable precision agriculture

**DOI:** 10.3389/fpls.2025.1706428

**Published:** 2026-01-14

**Authors:** Parvathaneni Naga Srinivasu, Aruna Pavate, G. JayaLakshmi, Jana Shafi, Jaeyoung Choi, Muhammad Fazal Ijaz

**Affiliations:** 1 Amrita School of Computing, Amrita Vishwa Vidyapeetham, Amaravati, Andhra Pradesh, India; 2 Department of Information Technology, Thakur College of Engineering and Technology, Mumbai, Maharashtra, India; 3 Department of Information Technology, Siddhartha Academy of Higher Education, Vijayawada, India; 4 Department of Computer Engineering and Information, College of Engineering in Wadi Alddawasir, Prince Sattam Bin Abdulaziz University, Wadi Alddawasir, Saudi Arabia; 5 School of Computing, Gachon University, Seongnam-si, Republic of Korea; 6 Centre for Artificial Intelligence Research and Optimization (AIRO), Design and Creative Technology, Torrens University Australia, Melbourne, VIC, Australia

**Keywords:** agentic AI, agriculture, crop monitoring, precision farming, SWOT analysis

## Abstract

**Introduction:**

Ensuring smarter and more sustainable farming practices is a critical challenge in modern agriculture. Agentic Artificial Intelligence (AAI), combined with Precision Agriculture (PA) and Federated Learning (FL), has the potential to enhance decision-making, optimize resource utilization, and reduce environmental impact.

**Methods:**

This study proposes an AAI based framework for precision agriculture that integrates distributed sensing devices, intelligent agents, and federated learning to enable real time monitoring and decision support at the farm level. A practical deployment architecture is outlined, detailing inter-device communication and localized intelligence. The proposed model is evaluated across two distinct datasets tomato disease classification and weed detection. The model is designed to have DenseNet121, MobileNetV2, EfficientDet-D0, and YOLOv8 as local models within a federated learning environment.

**Results:**

The federated global model achieved an accuracy of 96.4%, outperforming individual client models, with DenseNet121 and MobileNetV2 attaining accuracies of 95.0% and 93.9%, respectively. For weed species detection, EfficientDet-D0 demonstrated superior performance, achieving an mAP@0.5 of 0.978, average precision of 0.865, and an F1-score of 0.961, compared to YOLOv8 with an mAP@0.5 of 0.956 and an F1-score of 0.935.

**Discussion:**

The results confirm the feasibility and effectiveness of integrating AAI with federated learning for intelligent precision agriculture. A SWOT analysis highlights the strengths of the proposed approach, along with deployment challenges and constraints. Overall, this study establishes a roadmap for future research, emphasizing sustainable intelligent farming systems.

## 1 Introduction

Modern agriculture is undergoing a major transformation with the help of advanced technologies like federated learning (FL) (Naga et al., 2024b), agentic artificial intelligence (AAI) (Acharya et al., 2025), generative adversarial networks (GAN) (Lu et al., 2022), transfer learning (TL) (Das et al., 2024), and edge computing. These technologies work on intelligent data processing, real-time decision-making, and decentralized task modeling in farming environments. Among the most promising developments in recent times, AAI is much faster in the decision process, acts independently, and continuously learns from the environment. Unlike traditional AI, agentic AI systems are not just tools that follow predefined instructions; rather, they are capable of setting goals, adapting to changes, and working in complex, real-world situations (Murugesan, 2025). This makes them especially useful in fields like agriculture, where conditions are always changing, and quick decisions can make a big difference.

While AAI systems are capable of setting goals, their goal formation process in precision agriculture (PA) follows a well-defined, structured approach, which is a context-aware mechanism rather than following a predetermined decision-making process. In this domain, goals are typically derived from agronomic constraints, environmental signals, and predefined farm objectives. An irrigation agent could form a goal such as maintaining soil moisture within an optimal range based on crop requirements, by taking the weather predictions into consideration. This transition in farming model through AAI promises profound benefits like agricultural productivity and environmental sustainability. By precisely targeting available resource applications, AAI agents can dramatically reduce the impact of chemical fertilizers and pesticides on local water systems, mitigating pollution and preserving biodiversity. Furthermore, these AAI-driven systems can effectively identify and address crop diseases and pest infestations in much earlier stages, often resulting in better diagnosis and treatment, thereby preventing widespread crop loss and the need for broad-spectrum chemical treatments as adaptable.

Recently, PA has become a widely adopted approach in farming. It uses data, sensors, and automation to monitor and manage crops, soil, and other resources more accurately (Padhiary et al., 2024). The main focus is to provide the appropriate resources, such as water, fertilizer, or pesticide, at the right time and in precisely adequate amounts (Naga et al., 2024a). This not only assists in improving the crop yield but also reduces waste and minimizes environmental impact (Yousaf et al., 2023).

The transition to an AAI-driven farming model promises substantial benefits for both agricultural productivity and environmental sustainability. Conventional farming practices often lead to inefficient input usage, with studies reporting that 30%–50% of applied nitrogen and phosphorus fertilizers are lost through runoff or volatilization, contributing to soil degradation and water contamination. Likewise, broad-spectrum pesticide spraying can result in 20%–40% chemical drift, increasing ecological toxicity and affecting non-target organisms. AAI has the potential to mitigate these impacts by enabling precise, context-aware input management (Pimentel and Burgess, 2013). Through continuous sensing, localized decision-making, and autonomous task coordination, AAI systems can align fertilizer and pesticide application with actual crop needs, reducing unnecessary inputs and minimizing environmental leakage. Early evidence from precision agriculture implementations suggests that such AI-driven optimization can achieve 10%–25% reductions in chemical usage, depending on crop type, environmental variability, and data availability (Ding and Gao, 2025). Thus, the integration of AAI in precision agriculture represents not only an advancement in intelligent farming but also a quantifiable step toward more sustainable agricultural ecosystems. Furthermore, these AAI-driven systems can effectively identify and address crop diseases and pest infestations at their earliest stages, often resulting in better diagnosis and treatment, thereby preventing widespread damage that results in crop loss and the need for broad-spectrum chemical treatments that affect the environment. This not only secures food supply but also fosters healthier, more resilient ecosystems in farming.

**Figure 1:**
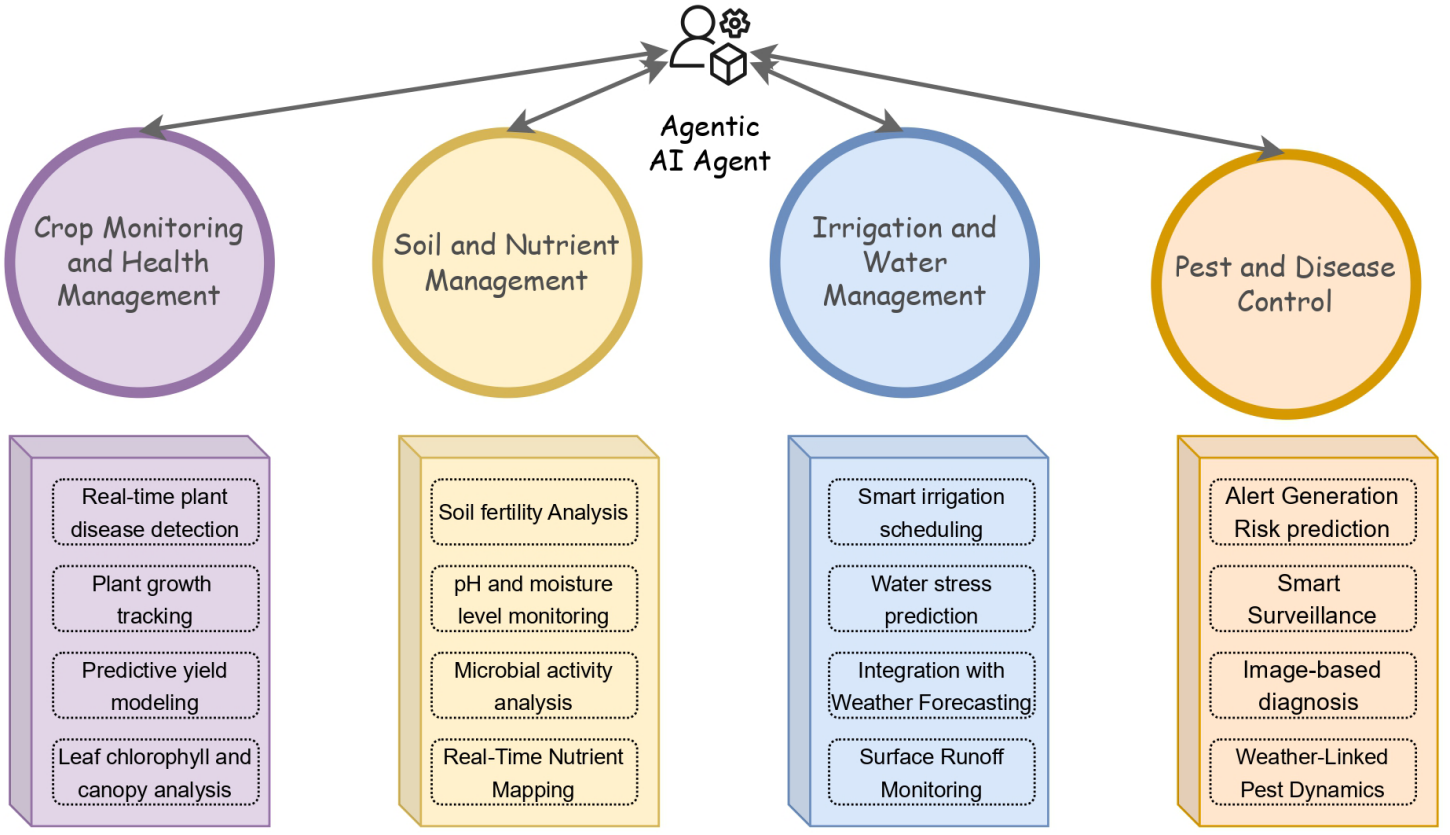
Various domains in precision agriculture that can be integrated with AAI technology.

In a smart precision farming environment, the use of multiple AI agents working together in a synchronized manner can significantly enhance the overall performance and efficiency of the models. Each agent can be assigned a specific task to perform tasks like monitoring soil moisture, detecting pests, analyzing weather patterns, and managing the irrigation (Zhang et al., 2024b). These agents operate independently but share information with each other in real-time, allowing for quick and informed decisions. When a pest detection agent identifies an early infestation, it can alert the nutrient and irrigation agents to ensure their operations accordingly, preventing crop stress and reducing chemical utilization. This kind of synchronization among the agents leads to timely actions, resource optimization, and reduced human intervention (Hosseini and Seilani, 2025). The collective intelligence and cooperation among agents create a more adaptive and responsive farming system, ultimately improving crop yield, conserving resources, and supporting long-term sustainability. **Figure 1** represents the various domains in PA where the AAI can be used to integrate them together.

Several studies have applied deep learning models such as Convolutional Neural Network (Dontha and Sri Supriyanka, 2023), DenseNet (Pillai et al., 2023), MobileNet (Zhang et al., 2024a), YOLO-based detectors (Dang et al., 2023), and EfficientDet (Cui et al., 2024) to crop-disease identification and weed detection, demonstrating strong baseline performance on curated image datasets. Prior research, however, has largely focused on isolated model development or centralized training settings, with limited attention to distributed learning, agent-driven decision frameworks, or their integration in agricultural intelligence systems. Existing works also seldom consider how perception models can operate as components within a broader autonomous, context-aware architecture capable of collaboration and decentralized reasoning. This manuscript addresses these gaps by situating model development within an agentic AI framework and employing federated learning to support distributed, privacy-preserving intelligence. The proposed approach advances beyond earlier studies by combining multilayer agentic design with empirical evaluation of both classification and detection tasks, thereby demonstrating how agent-based intelligence can be operationalized for real-world agricultural applications. The following are the contributions of the current study.

• The study outlines the agentic AI framework for precision agriculture, which outlines various key components of the architecture and how they are being integrated.
• The study presents a detailed framework on how AAI can be rolled out in PA; a case study has been included to analyze the impact of agentic AI in precision agriculture.
• The study discusses the strengths, weaknesses, opportunities, and threats of adopting AAI in PA.

While the proposed AAI framework has the potential to support more sustainable agricultural practices like improved decision-making process, optimized input use, and reduced crop losses, the anticipated benefits were not empirically validated within the scope of this study. The present work focuses on model development and federated learning evaluation using curated datasets and does not include the field-level measurements related to yield, sustainability indicators, and resource consumption. Future research will incorporate real-world trials to assess the practical impact of AAI-driven systems on yield improvement, input efficiency, and broader sustainability outcomes.

While the proposed agentic AI framework outlines the architectural components necessary for the autonomous decision-making process, the present study does not yet implement the full spectrum of agentic capabilities such as autonomous goal formation, long-horizon planning, reasoning-driven task decomposition, or multi-agent negotiation. Instead, the current work operationalizes the foundational layers like perception, distributed learning, and inference—upon which future agentic behaviors can be built. Thus, the empirical case study should be interpreted as demonstrating the enabling technologies that support agentic intelligence rather than a complete realization of an autonomous agentic ecosystem.

The rest of the manuscript is arranged as follows: the Background section discusses various fundamentals of AAI in precision agriculture. Section 3 is about the architecture and working mechanism of AAI in precision agriculture. Section 4 presents the SWOT analysis of the AAI technology. Section 5 is the conclusion and future research direction.

## 2 Background

The architecture of an AAI is designed to enable autonomy, adaptability, and collaborative decision-making in complex, real-world environments like farms. Unlike traditional rule-based systems, AAI operates through goal-driven agents that continuously sense, reason, act, and learn from their surroundings (Portugal et al., 2024).

• The perception layer is an interface layer that gathers real-time data in the physical environment using the sensors. It would assist the agents in situation assessment based on the dynamic conditions. The sensors could be the moisture sensors, soil pH sensors, salinity sensors, CC cameras, or satellite imagery that would gather the data and feed the raw data to the cognitive layer.
• In addition to collecting real-time environmental data, the perception layer includes a structured pipeline for integrating and managing heterogeneous sensors within the AAI system. Each sensing device, like the soil moisture monitoring probes, pH and salinity sensors, weather stations, CCTV cameras, satellite, and drone imagery, connects through IoT gateways and other edge nodes that perform essential preprocessing tasks. These include noise removal, data normalization, timestamp alignment, and basic anomaly filtering, ensuring that raw sensor readings are transformed into coherent and reliable data streams. The perception layer also incorporates a sensor orchestration module responsible for device registration, health monitoring, calibration checks, and fault detection, enabling the system to verify incoming data quality and request resampling whenever necessary. Processed data packets are transmitted to the cognitive layer using lightweight communication protocols, such as MQTT, LoRaWAN, and Wi-Fi, where they are combined with contextual information from the knowledge base. Through this integrated management framework, the perception layer ensures that the AAI system receives accurate, valid, and timely information, thereby improving the quality of situational assessment and subsequent decision-making process.
• The knowledge base is more than just a place to store information; it is also a structured entity that can grow into an information hub that helps all agents in the AAI system think and make decisions. Its internal structure usually has three parts, namely, a domain ontology that encodes agronomic concepts like crop types, soil attributes, disease profiles, nutrient requirements, and weather patterns; a spatiotemporal database that stores historical field measurements, sensor logs, and crop growth records; and a rule and policy layer that contains agronomic guidelines, threshold values, and operational constraints set by experts in the field and learnt from past interactions. Agents can get to the knowledge base through standardized query interfaces like API calls and ontology-based reasoning engines. These engines allow agents to find information that is relevant to the current task and takes into account the context. The knowledge base is always being updated in two ways: first, by automatically getting processed sensor data from the perception layer, and second, by the feedback that agents give after they do something. This dynamic update cycle makes sure that the knowledge base stays up to date with changing field conditions, which allows agents to improve their decisions and change their strategies over time. The knowledge base is the cognitive backbone of the AAI system because it is structured and interactive. It gives agents reliable, context-aware agricultural intelligence that they can use to make decisions.
• The cognitive layer is the part of the AAI system that makes decisions. Each agent uses the current state of the environment to process information, set goals, make plans, and take action. It uses adaptive strategies and reasoning algorithms to look at the options and choose the best ones. It would help figure out things like watering, getting rid of pests, and managing nutrients.
• The actuation layer is in charge of carrying out the tasks that the agents have decided on. It turns digital commands into actions in the real world on the farm. It connects to real-world devices like drones, irrigation systems, sprayers, and autonomous machines to do things like watering, fertilizing, and controlling pests with perfect timing and accuracy.
• The communication layer ensures seamless interaction and coordination among multiple agents, devices, and systems within the precision farming environment. It enables real-time data sharing, decision synchronization, and collaborative problem-solving, allowing agents to work together efficiently for tasks like scheduling irrigation and responding to pest outbreaks.

**Figure 2:**
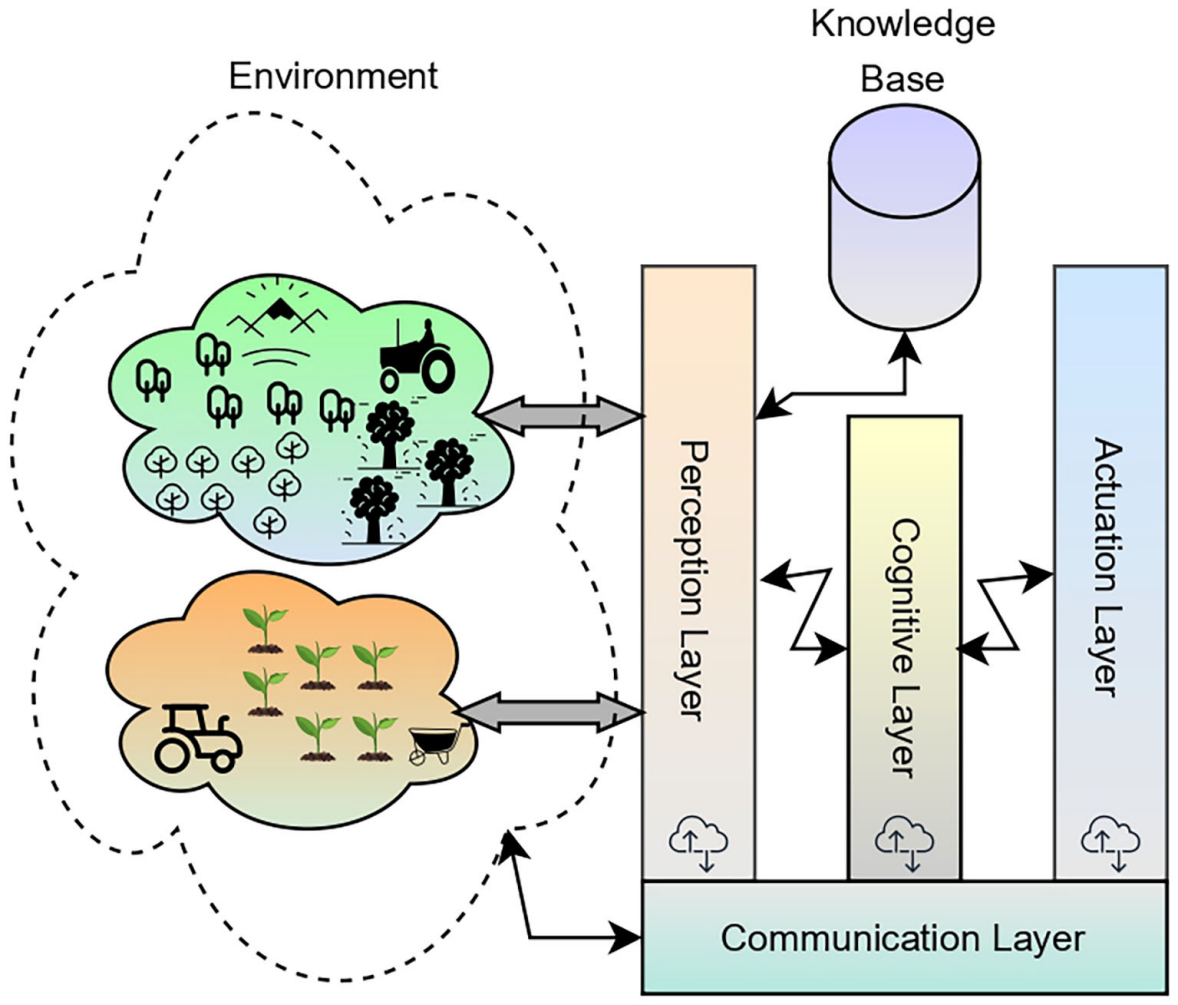
Image representing various components of the AAI model in precision agriculture.

The corresponding block diagram with all the major components of the AAI model is presented in **Figure 2**.

### 2.1 Learning process in AAI

The learning process in an AAI environment is through continuous interaction with the environment, where every agent observes, acts, and learns from the outcomes of its decisions. Agents begin by collecting real-time data through sensors, imagers, and other input devices (Pati, 2025). Based on these data, they make autonomous decisions like adjusting irrigation, recommending fertilizer, or signaling pest alerts. Then, it monitors the results of those actions. This feedback loop allows the agents to evaluate their choices and store the experience for future reference (Hughes et al., 2025).

In precision agriculture, the learning process within AAI agents is operationalized through well-established learning algorithms that enable continuous adaptation to field conditions. Reinforcement learning (RL) (Khosravi et al., 2025), for instance, is commonly implemented using algorithms such as Q-Learning, Deep Q-Networks (DQN), or Proximal Policy Optimization (PPO) to optimize tasks like irrigation scheduling, nutrient management, and pest intervention. An irrigation agent may use Q-Learning to identify optimal watering intervals by receiving rewards for maintaining soil moisture within target thresholds while minimizing water usage. Similarly, a crop health agent equipped with DQN can learn to detect early stress indicators in multispectral images, improving its diagnostic accuracy over time. Beyond RL, supervised learning models support perception tasks, while transfer learning and experiential learning allow agents to refine detection accuracy across varying field conditions. These algorithms operate within the feedback loop of sensing, acting, and learning, where agents update their policies based on environmental outcomes and long-term rewards. By integrating these learning methods, AAI systems become capable of autonomous optimization, enabling PA operations to adapt dynamically to crop needs and environmental variability. The corresponding algorithm is presented in **Algorithm 1**.

**Algorithm 1:**
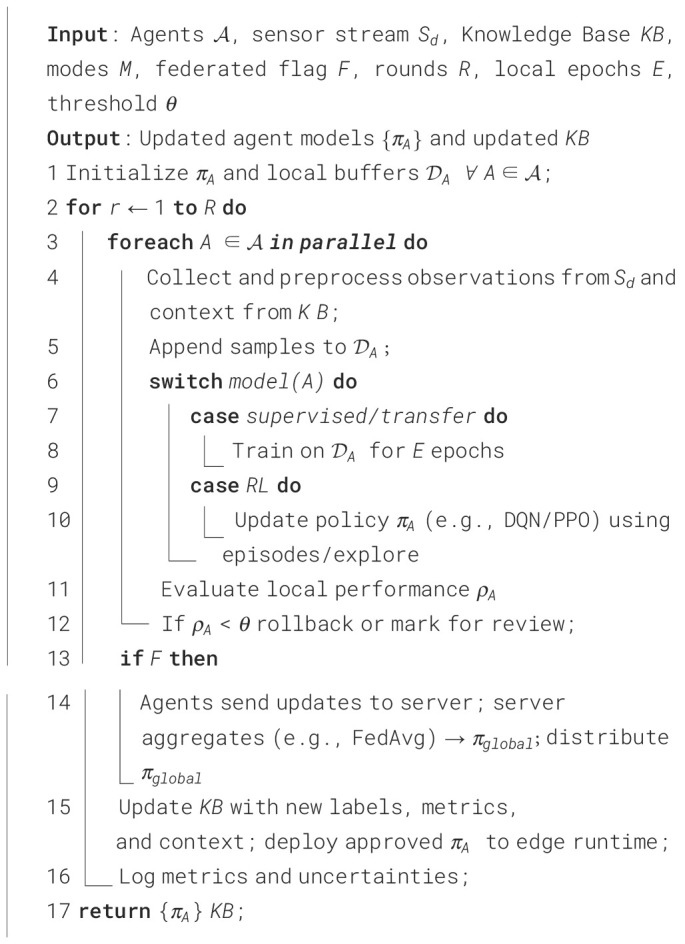
Concise learning process in Agentic AI.

## 3 Working mechanism of AAI in precision agriculture

**Figure 3:**
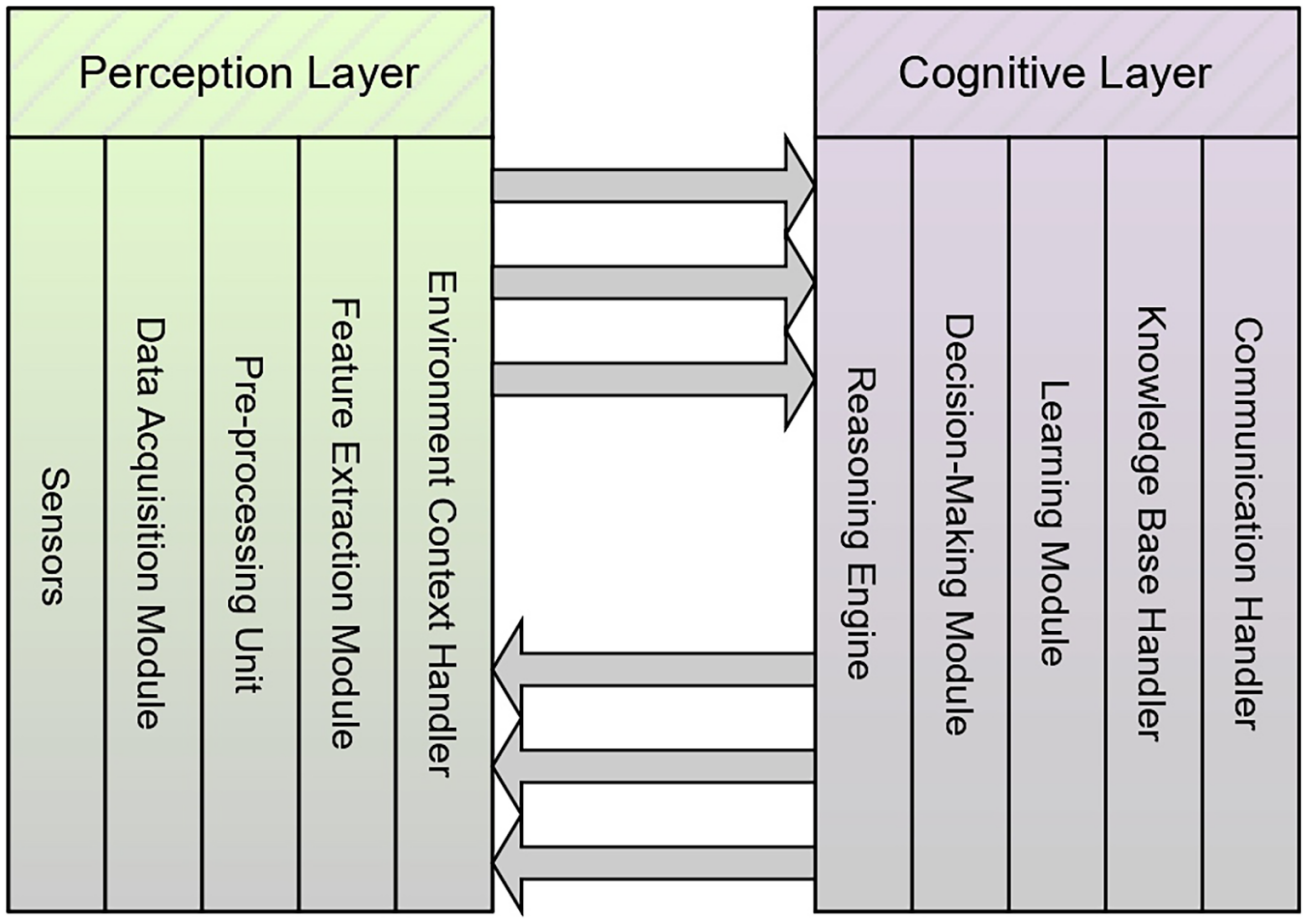
Image representing the communication between the perception layer and the cognitive layer.

The current section presents the working mechanisms and deployment procedures of the AAI model in PA. The three layers of the AAI model, which include the perception layer, cognitive layer, and actuation layer, are exceptionally significant in observing the environment, analyzing the context, and making a decision on the situation. The action was performed based on the decision that was made. The communication between the perception layer and the cognitive layer is shown using the following Algorithm 2 and the corresponding diagram in **Figure 3**.

**Figure 3** provides a visual interpretation of the workflow described in Algorithm 2 by illustrating how data pass through the perception layer to reach the cognitive layer. The algorithm outlines the procedure which involves data acquisition, preprocessing, event identification, and decision request generation. The figure presents these interactions as an integrated flow of information from different sensors into a unified perception module. In this diagram, sensor outputs converge into the perception layer, where contextual filtering and event triggering occur, mirroring the algorithm’s logic for determining when data exceed defined thresholds and require escalation. The arrows leading from the perception layer toward the cognitive layer visually represent the transmission of the decision request described in the algorithm, showing how processed sensor data become structured inputs for higher-level reasoning in the AAI system.

**Algorithm 2:**
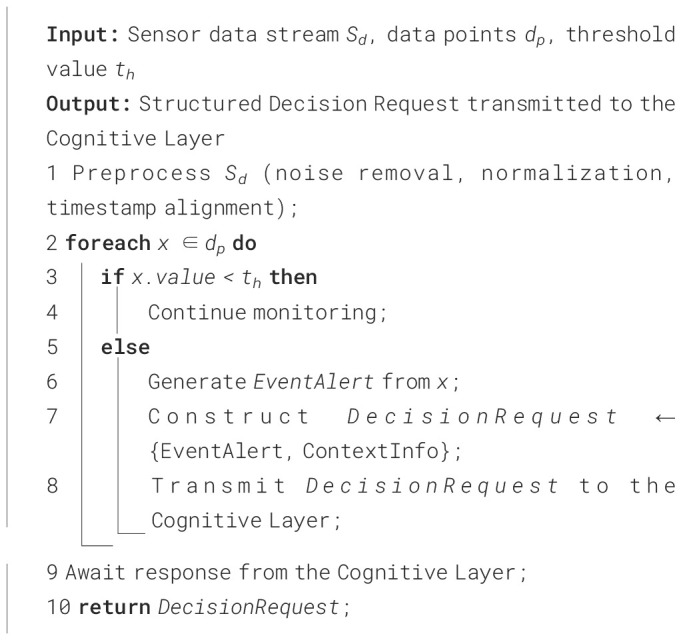
Communication between the perception layer and the cognitive layer.

**Figure 4:**
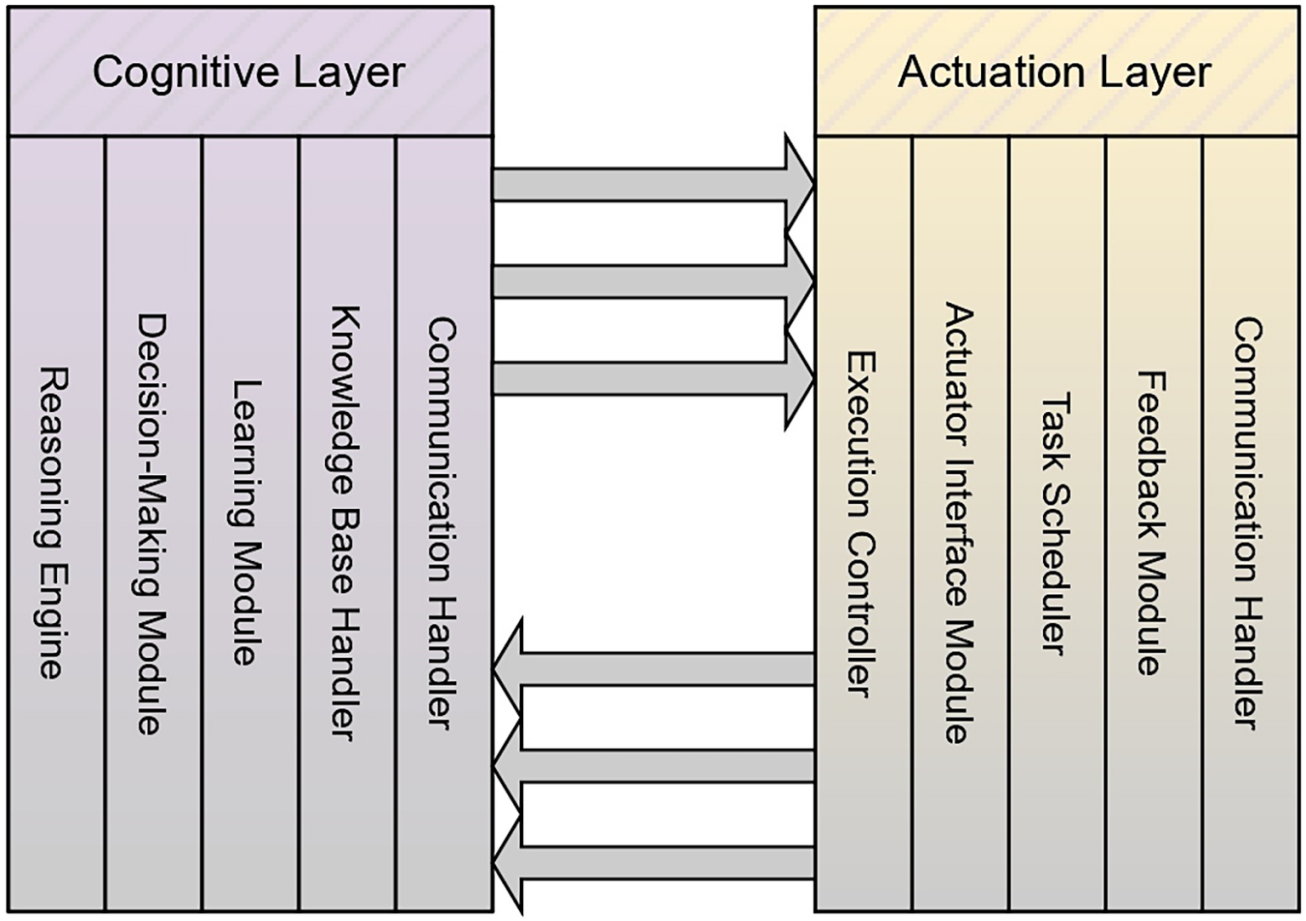
Image representing the communication between the cognitive layer and the actuation layer.

The communication between the cognitive layer and the actuation layer is significant in implementing the actions in the environment for the decisions that are made in the cognitive layer. The actuation layer is going to perform the actions for all the decisions that are made based on contextual information. The corresponding algorithm is presented in Algorithm 3, and the corresponding figure is presented in **Figure 4**. **Figure 4** complements **Algorithm 3** by visually depicting how the cognitive layer interprets incoming decision requests and translates them into executable actions within the farm environment. The algorithm describes the cognitive layer’s processes of reasoning, strategy selection, action-command generation, and feedback handling, and the figure presents these elements as interconnected components. The cognitive layer in the diagram is shown analyzing contextual information and issuing commands to the actuation layer through directional flows, reflecting the algorithm’s mechanism for selecting appropriate responses. The return pathway from the actuation layer back to the cognitive layer in the figure corresponds to the algorithm’s feedback-update cycle, highlighting how execution outcomes are reintegrated into the knowledge base to refine future decision-making. Together, the diagram and algorithm provide both procedural and structural perspectives on the interactions that drive autonomous action within the AAI framework.

**Algorithm 3:**
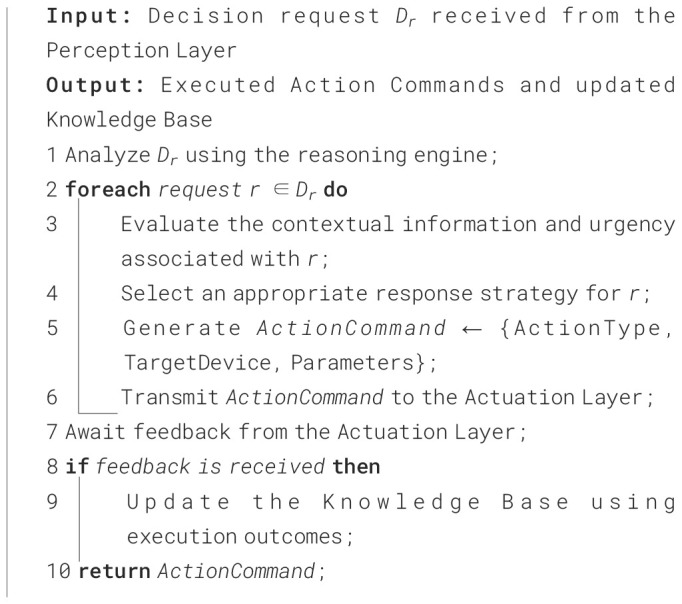
Communication between the cognitive layer and the actuation layer.

It can be observed from the above communication layout of the AAI model that the cognitive layer is crucial in perceiving, analyzing, and initiating action in the AAL environment. The working mechanism of the AAI model is explained using a deployment of AAI in PA as presented through a case study.

### 3.1 Agents in precision agriculture

**Figure 5:**
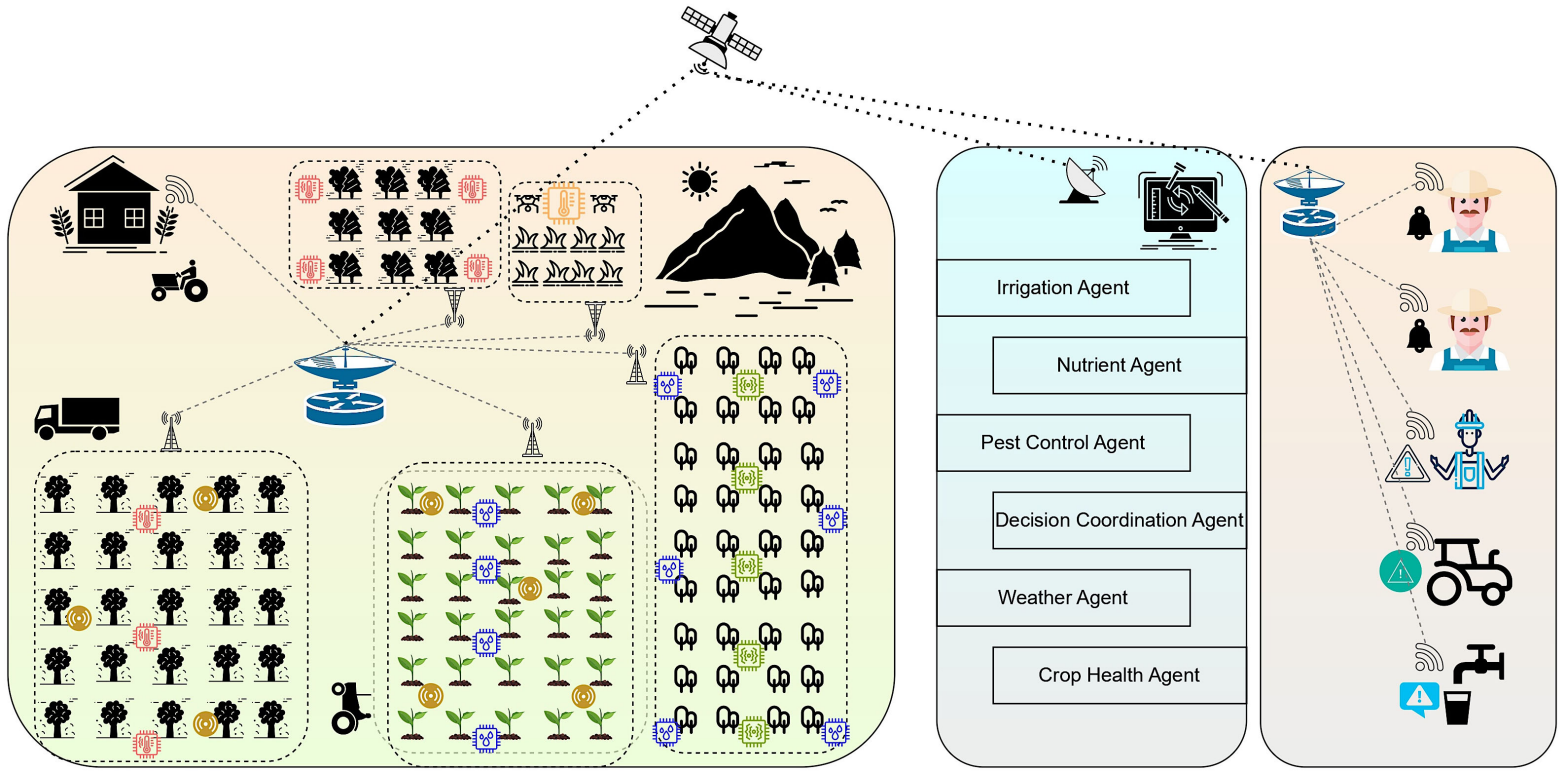
Image representing the communication between the cognitive layer and the actuation layer.

The PA environment would have multiple agents that would simultaneously work in collaboration with each other to make well-informed decisions. Some of the intelligent agents include irrigation, nutrient, pest control, and crop health agents that would be deployed across a medium-sized farm to monitor environmental conditions, make informed decisions, and coordinate timely actions (Nisa et al., 2025). These agents would improve the crop yield, optimize resource usage, and enable faster responses to changing field conditions through continuous agent collaboration. The image representing the crops, sensors, and base stations to collect the crop-related data is presented in **Figure 5**. The deployment of the AAI in the PA would have the following agents.

• The *irrigation agent* in PA is responsible for continuously monitoring the soil moisture content, weather conditions, and crop water requirements to optimize irrigation schedules. It would assist in making intelligent decisions on when, where, and how much water to apply, ensuring efficient water usage and thereby promoting healthy crop growth.
• The *nutrient agent* is responsible for assessing the soil nutrients and tracking the crop growth stages and environmental factors to determine the optimal timing and quantity of nutrient application. It ensures balanced fertilization, improving crop health while minimizing resource waste and environmental impact.
• The *pest control agent* detects signs of pest activity using data, imaging from the sensors, and understanding the environmental patterns to determine the appropriate action to be taken to control outbreaks. It enables the timely and targeted application of pesticides, reducing crop damage and minimizing chemical use in the process.
• The *decision coordination agent* would manage the synchronization among various agents, like the irrigation, nutrient, and pest control agents, to ensure a unified and conflict-free decision-making process. It also assists in prioritizing actions and maintaining consistency across all farming operations that are performed for better crop management.
• The *weather agent* collects and analyzes real-time meteorological data to predict weather patterns that may affect farming activities and crop growth. It sends timely alerts and guidance to other agents, helping optimize decisions related to irrigation and fertilization based on upcoming weather conditions.
• The *crop health agent* would continuously monitor the plant conditions using sensor data, imaging, and growth patterns to detect signs of stress or nutrient deficiencies. It provides timely insights and recommendations to other agents that enable early intervention and promote better crop development.

All these agents would work synchronously and alert the farms and automated agents to respond promptly with actions like watering, fertilizing, or pest control, leading to more efficient and proactive farm management in a timely manner.

## 4 Case study

This case study would explore the practical application of agentic AI in a real-world PA setting. It is important to clarify that the implemented models in the case study serve primarily as the perceptual and inference modules within the broader agentic architecture. They provide the essential building blocks—classification, detection, and federated aggregation—from which higher-order agentic capabilities such as self-directed planning, goal generation, and coordinated multi-agent reasoning can emerge in future extensions. Therefore, the case study is not presented as a full demonstration of autonomous agentic behavior, but rather as a proof-of-concept illustrating how perception-level intelligence integrates into the larger AAI framework. In the current study, the case study is performed with four agents that are deployed in an FL environment (Dembani et al., 2025), where two local agents are designated to perform disease detection from the leaf as well as two agents to perform weed detection from the farmland. The case study is evaluated over the tomato crop for diagnosing the abnormalities and timely intervention. The details of the local models that are used in the current study are presented in **Table 1**. The comparison of the FL with other technologies like edge (Pintus et al., 2025) and fog (Boukhali et al., 2025) is presented in **Table 2**.

**Table 1:** Agents and local models used in the current study.

Agent	Task	Dataset	Architecture	Purpose
Leaf disease	Agent 1	Tomato leaf dataset	MobileNetV2 (Duhan et al., 2025)	Tomato leaf disease classification on edge devices
Leaf disease	Agent 2	Tomato leaf dataset	DenseNet121 (Raza et al., 2025)	High-accuracy tomato leaf disease classification from image data
Weed detection	Agent 3	Crop–weed dataset	YOLOv8 (Liu et al., 2024)	Smart weed and crop classification model from image data
Weed detection	Agent 4	Crop–weed dataset	EfficientDet-D0 (Ma et al., 2025)	Lightweight object detection model for weed detection from aerial images.

**Table 2:** Comparison of edge, fog, and federated learning in precision agriculture.

Feature	Edge computing	Fog computing	Federated learning
Low latency	✓	✓	•
Real-time processing	✓	✓	✗
Bandwidth efficiency	•	✓	✓
Scalability	✗	•	✓
Energy efficiency	✓	•	•
High data privacy	•	•	✓
Offline functionality	✓	•	✗
High computational power	✗	✓	•
Cost effectiveness	✓	•	•
Collaborative model training	✗	✗	✓
Cross-farm knowledge sharing	✗	•	✓
Data sharing restrictions	•	•	✓
Adaptability to heterogeneous devices	•	•	✓
Model personalization	✗	•	✓

✓ indicates strong support, ✗ indicates not supported, and • indicates partial support.

The datasets selected for the case study were chosen because they represent common, high-impact challenges frequently encountered in real-world precision agriculture systems. The tomato leaf disease dataset includes diverse visual conditions, multiple disease categories, and variations in lighting, leaf orientation, and background noise, closely mirroring field environments where imaging conditions are rarely uniform. This makes it suitable for evaluating the robustness of classification agents intended for on-field disease monitoring. Similarly, the weed crop dataset reflects practical agricultural scenarios by incorporating images captured from real farm plots with mixed crop and weed distributions, irregular weed growth patterns, and heterogeneous soil textures. These characteristics simulate the visual complexity faced by weed detection agents deployed on drones or ground robots. Together, these datasets provide a realistic benchmark for testing the perception and learning capabilities of the AAI system, ensuring that the findings generalize beyond controlled laboratory conditions and remain applicable to operational PA settings.

The case study presents the fundamental learning components of an agentic system rather than a full demonstration of autonomous agency. The implemented models supply the perception and inference capabilities on which future agentic behaviors will be built. The implemented models primarily provide perception and inference capabilities, such as the local decision support through classification, detection, and federated learning, that serve as the essential building blocks for more advanced agentic behaviors. These components establish the informational and computational baseline structure upon which future extensions, including autonomous goal formation, multi-agent coordination, planning, and self-directed adaptation, can be developed.

### 4.1 Dataset description

The current study has used two distinct datasets: one for leaf disease prediction and the other for weed detection in the farmland. The details of the dataset are listed below.

#### 4.1.1 Dataset 1

**Figure 6:**
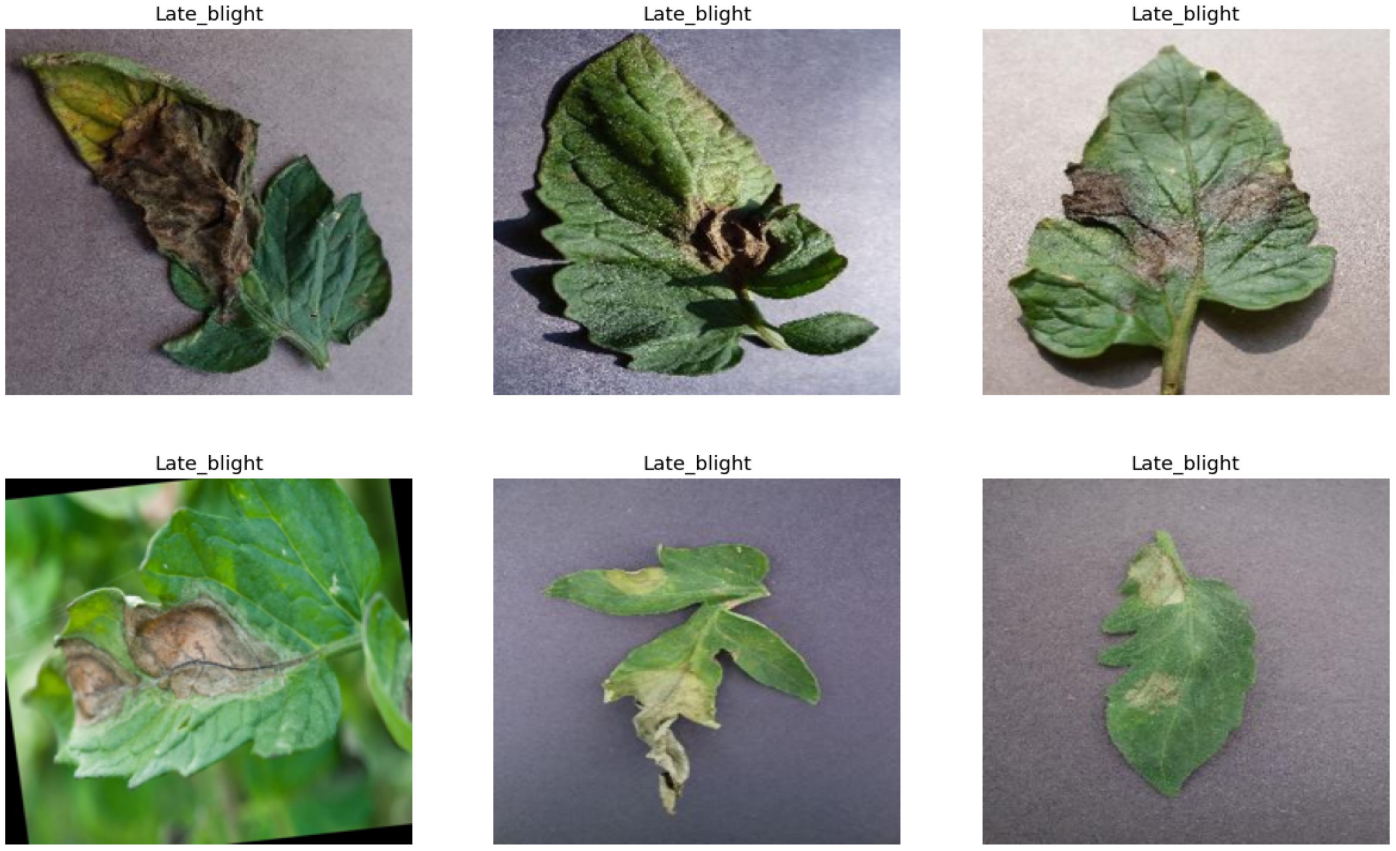
Sample images in the plant leaf disease detection dataset.

The first dataset corresponds to the leaf disease-related dataset, i.e., the Tomato Leaf Dataset (Ashish Motwani, 2025), which is an open-access dataset from Kaggle. The dataset is an openly accessible dataset with 32,534 samples of images across 11 classes. The dataset consists of various types of leaf diseases which include late blight with 3,905 samples, *Septoria* leaf spot with 3,628 samples, bacterial spot with 3,558 samples, leaf mold with 3,493 samples, early blight with 3,098 samples, tomato mosaic virus with 2,737 samples, tomato yellow leaf curl virus with 2,537 samples, target spot with 2,284 samples, spider mites (two-spotted spider mites) with 2,182 samples, and powdery mildew with 1,256 samples, and the rest of the 3,856 samples are of healthy leaves. All the images are in JPEG format, and each image has a size of 256 × 256. The samples of the dataset are shown in **Figure 6**. The number of samples in the dataset that are used for the training and validation is presented in **Table 3**. The testing samples are acquired from the validation samples with a ratio of 50%.

**Table 3:** Class-wise distribution of training and validation samples of the tomato leaf disease dataset.

Class	Training samples	Validation samples
Late blight	3,113	792
*Septoria* leaf spot	2,882	746
Bacterial spot	2,826	732
Leaf mold	2,754	739
Early blight	2,455	643
Tomato mosaic virus	2,153	584
Tomato yellow leaf curl virus	2,039	498
Target spot	1,827	457
Spider mites	1,747	435
Powdery mildew	1,004	252
Healthy	3,051	805

#### 4.1.2 Dataset 2

**Figure 7:**
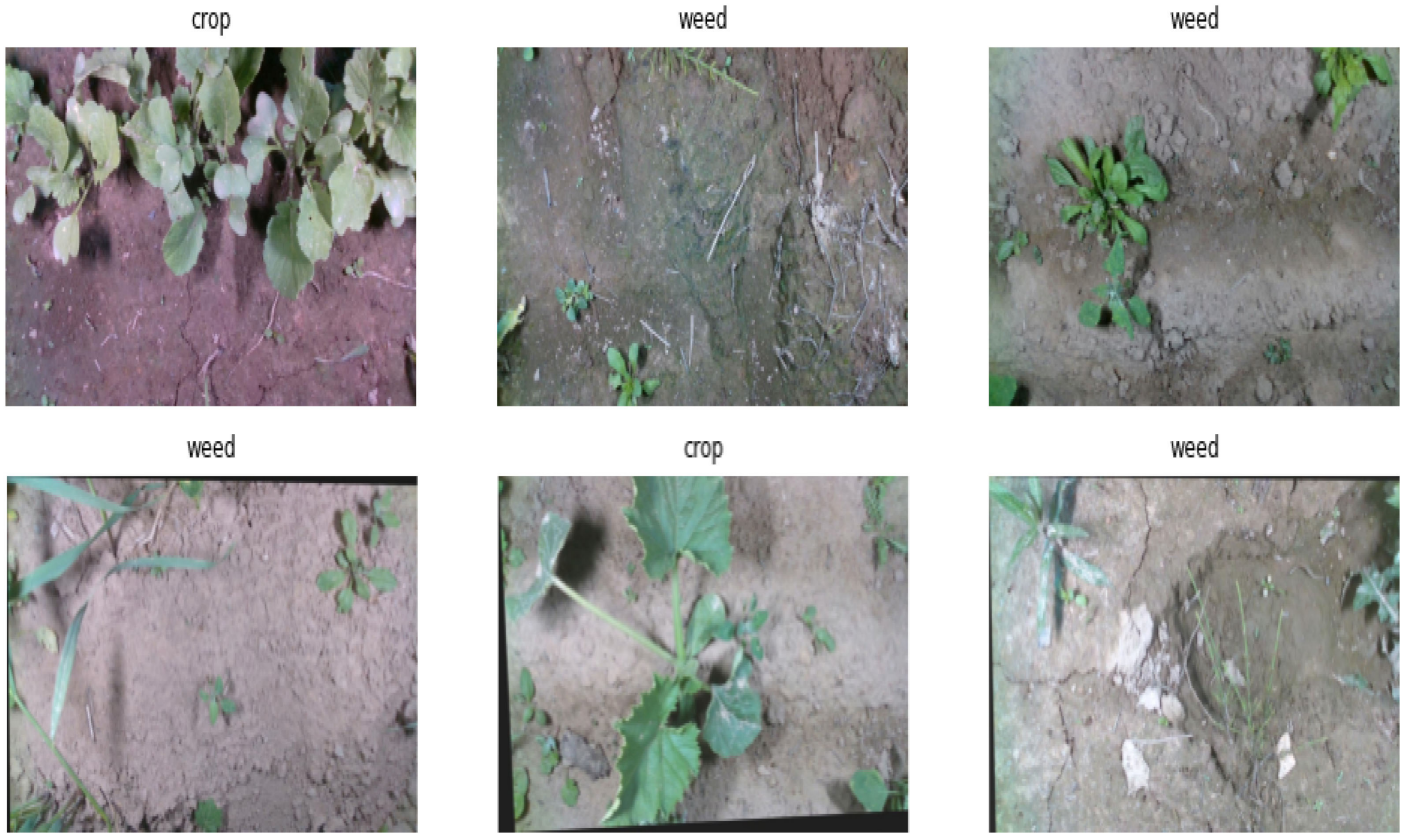
Sample images from the WeedCrop image dataset.

The second dataset corresponds to the weed detection dataset, i.e., the WeedCrop Image Dataset (Sudars et al., 2020), which is an open-access dataset from Kaggle. The dataset consists of 2,822 images of both crop and weed classes (Shanawad, 2025), out of which 2,469 samples are used for training the model, 235 samples are used for validation purposes, and 118 samples are used for testing purposes. The splitting of the data samples comes with the dataset, and no specific strategy is followed in the current study for splitting the dataset. The image dataset is augmented with rotation, shear, and random brightness adjustment. The images are acquired from six food crop species and eight weed species. The images vary in size and in JPEG format. The sample images of WeedCrop Image dataset are presented in **Figure 7**.

To ensure the integrity and reliability of the experimental results, necessary measures were taken to prevent data leakage. The dataset was partitioned into training, validation, and testing subsets using stratified, non-overlapping splits, and all preprocessing steps were only performed on the training samples. In addition, in the federated learning setup, each client got separate partitions, making sure that no image overlapping has occurred. These steps make sure that the reported model performance is an appropriate measure of its ability to generalize the data.

### 4.2 Hyperparameters and experimental settings

The current section presents the hyperparameters that are considered at each local model. Hyperparameter tuning is not performed in the current study, and the parameters are decided based on the default settings that are used with each of the models. The details of the hyperparameters used in each model are presented in **Table 4**.

**Table 4:** Hyperparameters used for each local model in experimental evaluation.

Hyperparameter	MobileNetV2	DenseNet121	YOLOv8	EfficientDet-D0
Optimizer	Adam	Adam	SGD	AdamW
Learning rate	0.0002	0.0001	0.01	0.0002
Batch size	32	32	16	16
Number of epochs	50	50	100	100
Loss function	Categorical cross-entropy	Categorical cross-entropy	CIoU + BCE + Obj Loss	Focal loss
Dropout rate	0.3	0.4	–	–
Input image size	224 × 224	224 × 224	640 × 640	512 × 512
Early stopping patience	10	10	20	15
Verbose	1	1	–	–
Weight decay/L2	0.0001	0.0001	0.0005	0.0001

The proposed model was experimented with in a remote cloud environment using Google Colab with GPU acceleration enabled. The experimental setup included an NVIDIA Tesla T4 GPU, 16 GB RAM, and a runtime environment using Python 3.10. The federated learning setup was simulated by partitioning the dataset into multiple non-overlapping subsets, representing independent clients. Each client performed local training for a fixed number of epochs using its assigned data, after which the locally updated weights were securely aggregated on the server using the federated averaging (FedAvg) algorithm (Xing et al., 2022). The FL setup in this study was designed to reflect realistic distributed agricultural environments where data sources naturally differ. In the current study, the global model is built from four local models that are connected. The configuration details of the FL model are discussed in **Table 5**.

**Table 5:** Configuration used in the federated learning environment.

Parameter	Configuration
Number of clients	4
Partitioning strategy	Non-IID
Communication rounds	20
Local epochs per round	5 epochs per client
Aggregation method	FedAvg

### 4.3 Global model

In FL settings, the global model is not trained directly on centralized data; instead, it is constructed iteratively through the aggregation of model parameters from distributed client devices. Each client maintains an identical model architecture with two heads, where one is dedicated to leaf disease classification and the other to weed detection. During each federated communication round, clients perform local training using their private, non-overlapping data partitions, updating both the shared feature extractor and the task-specific heads.

The global model employs a multitask architecture in which a shared convolutional backbone based on DenseNet121 is used to extract low-level and mid-level visual features that are relevant to both disease classification and weed detection tasks. DenseNet121 was selected due to its densely connected layers, which facilitate strong feature reuse and efficient gradient propagation, enabling the model to learn rich representations even with heterogeneous client data. On top of this shared backbone, two task-specific heads are integrated. The classification head consists of a global average pooling layer, followed by fully connected layers and a softmax output layer to predict the 11 disease categories, optimized using categorical cross-entropy (CCE) loss. The detection head, inspired by the YOLOv8 design, incorporates convolutional prediction layers that generate bounding box coordinates, objectness scores, and class probabilities using complete intersection over union (CIoU) and binary cross-entropy (BCE)-based losses. These individual layers in the architecture allow the backbone to learn generalized visual patterns from multiple tasks, while each head focuses on the specialized objectives required for its domain. By aggregating feature updates from distributed clients through federated averaging, the global model benefits from diverse field patterns, resulting in improved generalization and enhanced performance across both classification and detection tasks.

Once local training is completed, only the updated model weights and bias values are transmitted to the central server—raw data never leave the clients. The server then applies the FedAvg algorithm, which computes a weighted average of the client updates based on the size of each client’s dataset. This aggregated parameter set forms the updated global model, which is redistributed to all clients for the next round of local training. Over multiple communication rounds, the global model converges toward a representation that generalizes across the distributed data while preserving client privacy.

To effectively handle both leaf disease classification and weed detection, the global model uses a shared convolutional backbone over DenseNet121 for feature extraction, followed by two dedicated task heads. The classification head employs a softmax activation function to output class probabilities across all disease categories and is optimized using the categorical cross-entropy loss. The softmax activation in the output layer would produce the probability distribution across all the classes. The classification loss is defined as shown in Equation 1.

(1) $$L_{\text{cls}}=-\sum_{c=1}^{max}y_{c}\text{log}({\hat{y}}_{c})$$

**Figure 8:**
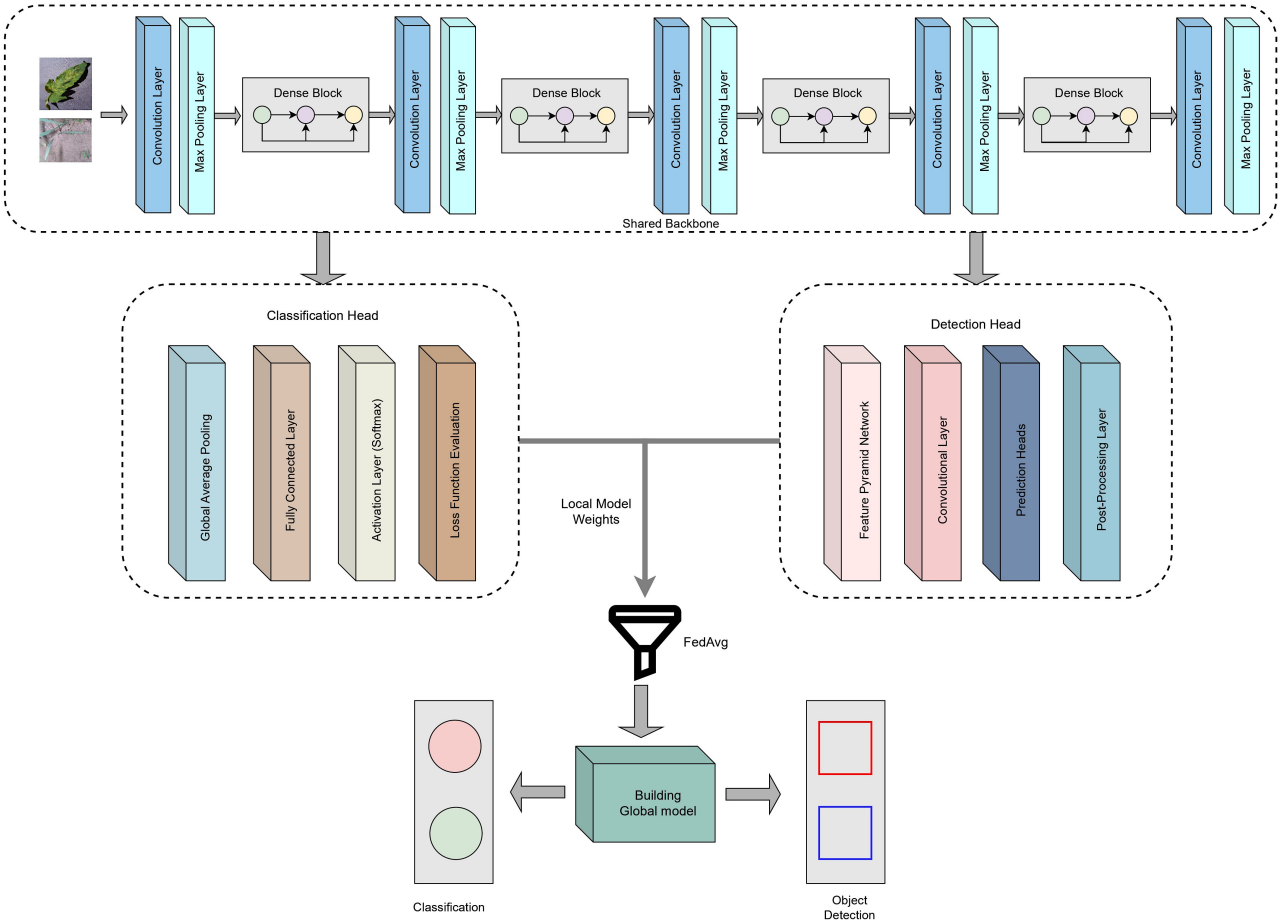
Architecture of the global model with multiple heads over the shared backbone.

From the equation, the notation *c* represents the number of classes, and the notation L_cls_ represents the classification loss. *y_c_* designates the ground-truth associated with the class *c* and ${\hat{y}}_{c}$ designates the predicted probability for class *c*. The weed detection head follows a YOLOv8-style detection head, producing bounding boxes and objectness scores, and is trained using a composite detection loss function comprising CIoU loss for bounding box regression, BCE loss for objectness prediction, and BCE loss for class probabilities. The corresponding figure of the global model with two heads is shown in **Figure 8**.

To emulate real-world variations across farms, the client datasets were constructed using a non-independent and identically distributed (non-IID) partitioning strategy. Non-IIDness was primarily introduced through label imbalance, where each client received samples from only a subset of disease classes rather than the full distribution. This simulates practical scenarios in which certain farms encounter only specific diseases depending on climate, crop type, and seasonal patterns. The number of samples assigned to each client was varied to represent the uneven data availability typically seen across real farms. No manual feature-distribution skew was introduced; however, natural differences in lighting, leaf appearance, and background conditions within the dataset contributed to feature diversity. Together, these factors created realistic non-IID conditions, enabling assessment of the robustness and adaptability of the proposed federated agentic framework under practical, uneven data scenarios.

### 4.4 Multitask optimization in federated learning

The global model uses DenseNet121 as a shared backbone with two task-specific heads: a classification head for tomato leaf disease prediction and a detection head for weed localization (bounding box regression). During each local training cycle, both heads produce task-specific losses, which are combined into a single scalar objective as shown in Equation 2.

(2) $$L_{\text{total}}=\lambda_{\text{cls}}L_{\text{cls}}+\lambda_{\text{det}}L_{\text{det}}$$

From the above equation, the notation $L_{\text{cls}}\;$ is the categorical cross-entropy loss and $L_{\text{det}}$ designates the sum of CIoU, BCE-objectness, and BCE-classification losses from the detection head. In the current study, both weights $\lambda_{\text{cls}}=\;\lambda_{\text{det}}=1$ giving equal importance to both tasks. This single combined loss is used to update both the shared backbone and task heads on each client.

During aggregation, the server applies FedAvg to the complete parameter set of the shared backbone and the two heads. FedAvg averages the parameter updates without needing explicit gradient balancing at the server. Although multitask training can lead to gradient conflict between objectives, no divergence was observed in our experiments, likely because classification and detection rely on overlapping visual features.

### 4.5 Performance analysis

**Figure 9:**
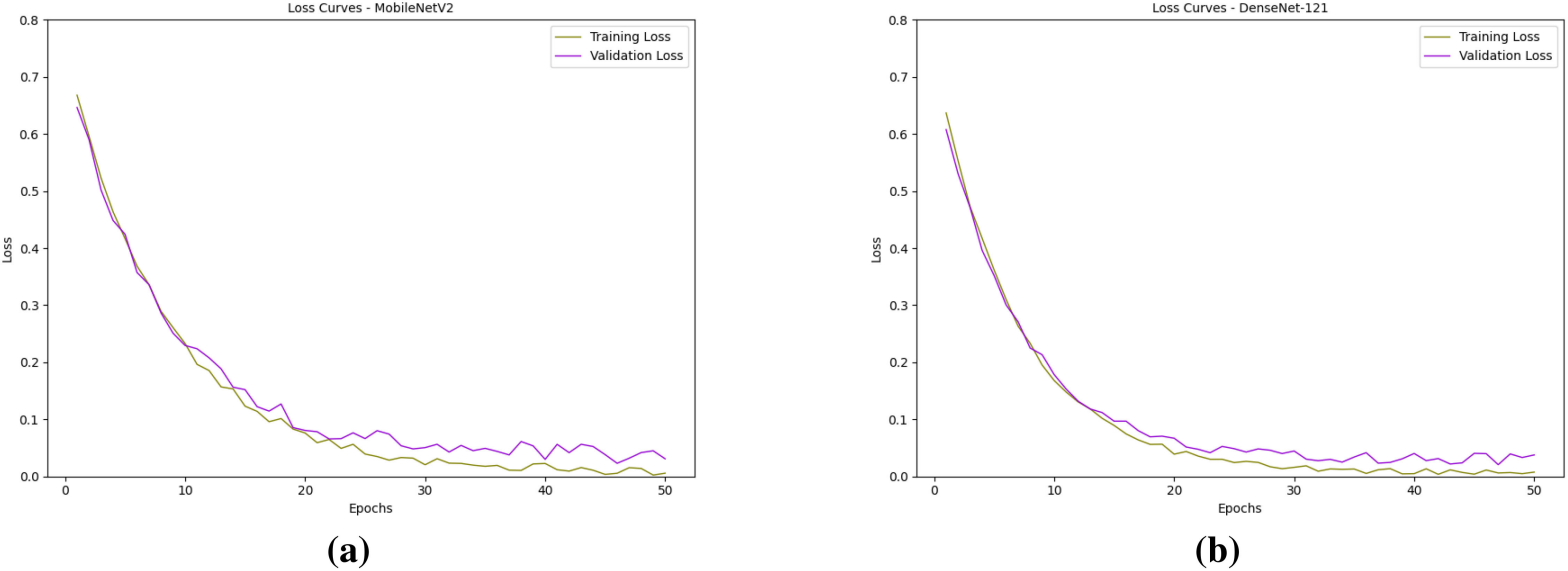
Observed loss curves of the models: **(a)** MobileNetV2 and **(b)** DenseNet121.

**Figure 10:**
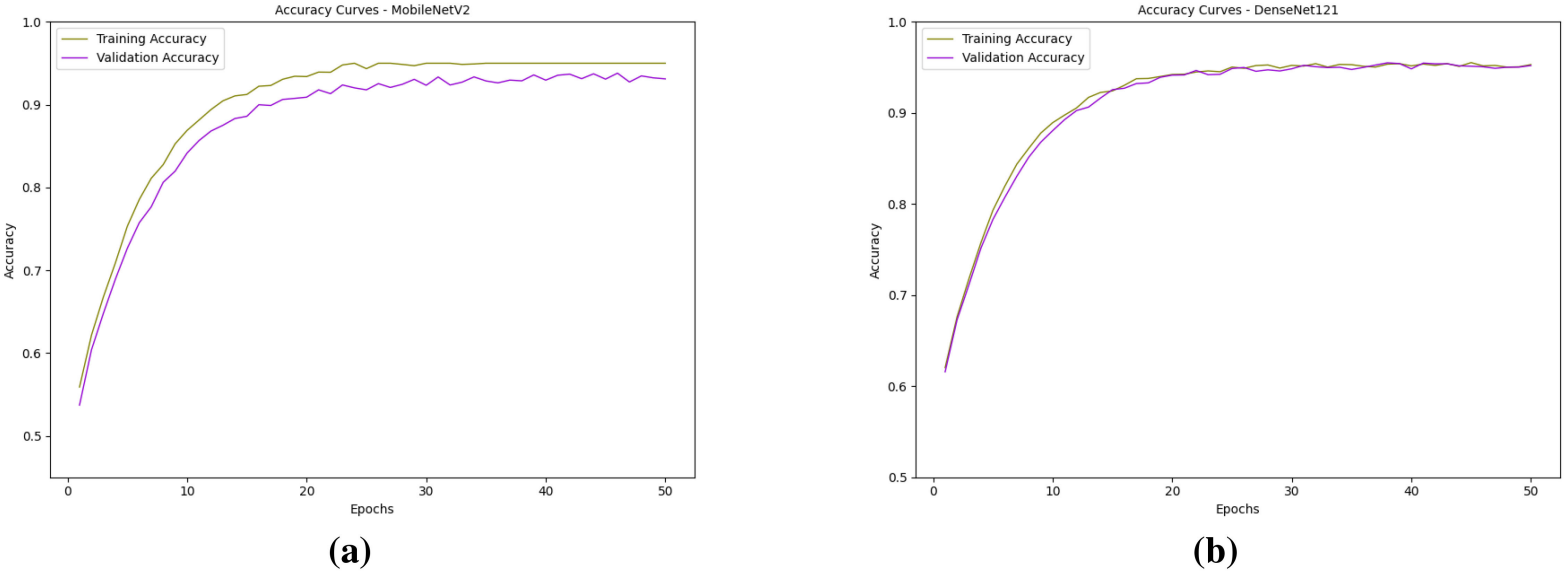
Observed accuracy curves of the models: **(a)** MobileNetV2 and **(b)** DenseNet121.

The performance of all local models is being evaluated in relation to the model learning process and the building process using loss and accuracy graphs (Sekhar et al., 2022). For the classification models, training and validation losses and accuracies were monitored across epochs, enabling the assessment of model stability, detecting scenarios like overfitting and underfitting, and estimation of generalization capability to unseen data. The observed loss graphs of both the local models are presented in **Figure 9**, and the accuracy graphs are presented in **Figure 10**.

**Figure 11:**
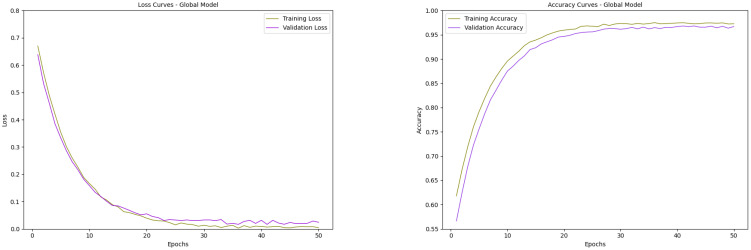
The loss and accuracy graphs of the global model.

Both MobileNetV2 and DenseNet121 demonstrated strong convergence and generalization performance during training. The loss curves for both models show a steady decline, with training and validation losses closely following each other, indicating minimal overfitting. DenseNet121 exhibited slightly smoother convergence with consistently lower validation loss in the later epochs, reflecting its ability to learn more robust representations. In contrast, MobileNetV2 showed slightly higher fluctuations in validation loss but still achieved a low final loss, suggesting good generalization. Accuracy curves for both models indicate significant improvement within the first 15–20 epochs, after which they gradually plateau. DenseNet121 achieved marginally higher validation accuracy and maintained a closer match between training and validation accuracy across epochs, showing superior stability. Overall, while both models performed well, DenseNet121 slightly outperformed MobileNetV2 in terms of final validation accuracy and consistency, making it a more reliable choice for this task. The performance of the global model with respect to the classification head is presented in **Figure 11**.

**Figure 12:**
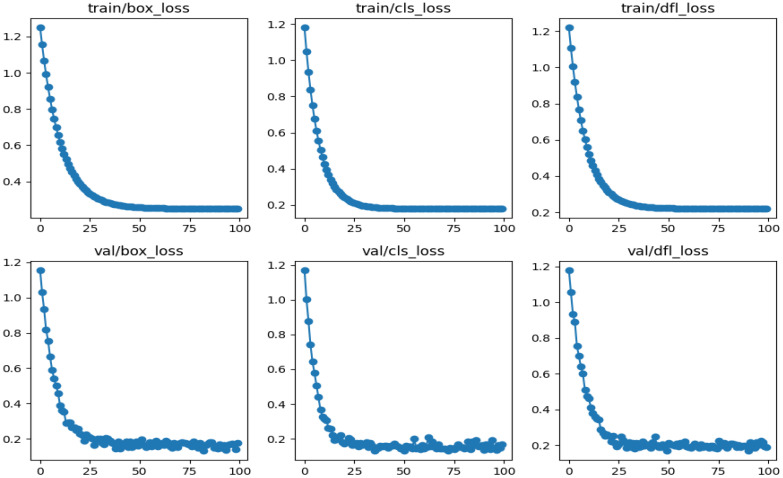
Graphs representing the loss curves associated with the YOLOv8 model.

The performance of the weed detection model using YOLOv8 and EfficientDet-D0 is evaluated in terms of the loss measures like class loss, box loss, and distribution focal loss, which would assist in assessing the generalizability of the model. The box loss measures the accuracy of predicted bounding box locations compared to the ground truth, directly reflecting how well the model is capable of localizing the object. Classification loss assesses the correctness of the predicted class labels, ensuring precise classification of object categories. Distribution focal loss further refines bounding box regression by modeling the distribution of bounding box offsets, thereby improving boundary alignment precision. Tracking these losses throughout training provided insights into the models’ ability to accurately localize weeds and maintain high-confidence predictions under diverse field conditions. The loss values associated with the YOLOv8 model are presented in **Figure 12**.

**Figure 13:**
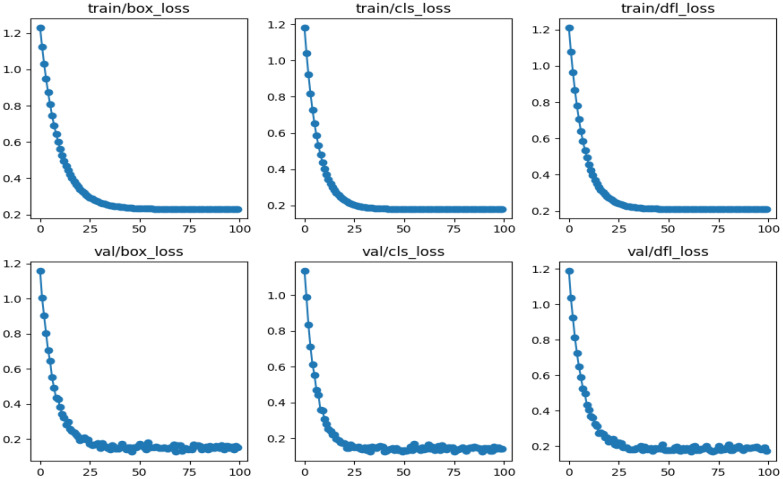
Graphs representing the loss curves associated with the EfficientDet-D0 model.

The loss values measures for the EfficientDet-D0 model are being evaluated and have shown a reasonable performance. The corresponding loss graphs are presented in **Figure 13**. The performance of the EfficientDet-D0 model in weed detection demonstrated clear improvements over the YOLOv8 model when evaluated using box, classification, and distribution focal loss measures. EfficientDet-D0 achieved lower box loss, indicating more precise localization of weed regions while also attaining reduced classification loss, which reflects higher reliability in distinguishing weeds from non-weeds. Additionally, the model showed a consistent reduction in decentralized federated learning (DFL), highlighting its ability to better align bounding box distributions with ground-truth annotations. These results collectively suggest that EfficientDet-D0 provides a more balanced and generalizable detection framework compared to YOLOv8.

### 4.6 Potential limitations

This study’s overall framework is based on AAI; however, the case study focuses on the perception and learning parts, which include supervised classification, detection, and federated aggregation. These elements constitute the essential competencies necessary for agentic systems; nevertheless, they do not currently facilitate autonomous goal creation, multi-agent negotiation, planning, or self-directed task breakdown. Therefore, the case study should be viewed as a proof-of-concept for data-driven local intelligence within the larger AAI architecture, rather than a comprehensive demonstration of agentic behaviors. All evaluations are conducted on Kaggle datasets, which are controlled and curated. One of the major potential limitations of the current study is that it cannot account for the dynamic conditions like the real agricultural environments, which have substantial variability, including inconsistent lighting, occlusions caused by the leaves or wind, overlapping disease symptoms, sensor noise, soil and humidity fluctuations, and crop-specific growth patterns.

The current study reports results using standard metrics for each model configuration. Although these results provide useful insights into model behavior, the use of repeated runs, variance measurements, and statistical analysis was not incorporated at this stage. Including such procedures in future work would help strengthen the robustness of the findings and provide a clearer understanding of performance stability across different training executions. The current study does not include ablation analyses on factors such as the number of clients, backbone architecture, and non-IID data distribution settings that would assist in better comprehensibility of the model’s abilities.

## 5 Experimental analysis

**Figure 14:**
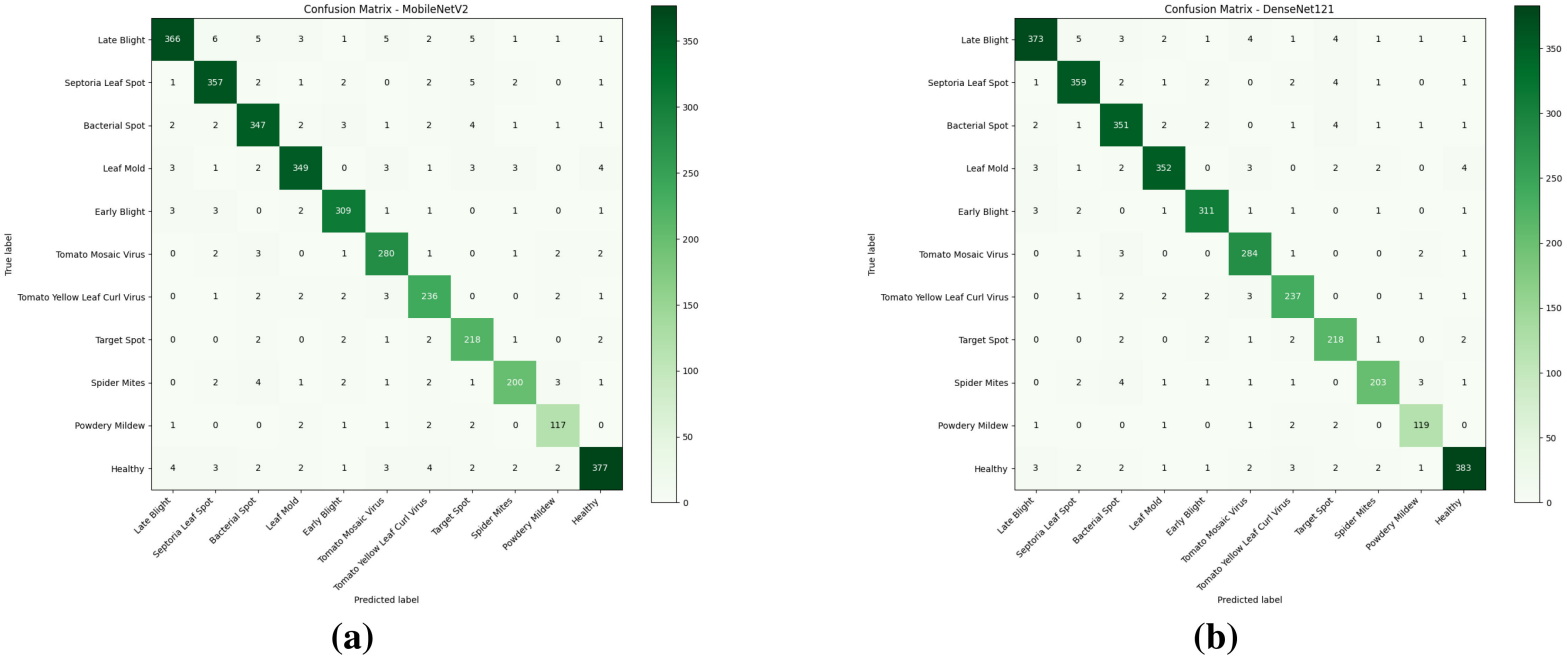
The observed confusion matrices of the local models: **(a)** MobileNetV2 and **(b)** DenseNet121.

The performance of the proposed model was evaluated using standard evaluation metrics. The classification and detection outcomes of all the local models and the global model were assessed. The classification models were evaluated with respect to standard metrics such as precision, recall, F1-score, accuracy, and specificity. Precision measures the proportion of correctly identified positive cases among all predicted positives, indicating the model’s ability to minimize false positives. Recall quantifies the proportion of actual positives correctly identified, highlighting the model’s effectiveness in capturing true cases. The F1-score provides a harmonic mean of precision and recall, offering a balanced measure when both metrics are equally important. Accuracy represents the overall proportion of correctly classified instances among all samples. Specificity measures the proportion of correctly identified negative cases, reflecting the model’s ability to avoid false alarms. Together, these metrics provide a comprehensive evaluation of the model’s performance across different aspects of classification. The result of the classification of the first two local models is presented in the confusion matrices in **Figure 14**, and the values of various metrics are presented in **Table 6**. The model’s performance across all multiclass metrics was computed using macro averaging, treating each class equally regardless of sample size.

**Table 6:** Performance of DenseNet121 and MobileNetV2 on tomato leaf disease classification.

Model	Precision	Recall	F1-score	Accuracy	Specificity
DenseNet121	0.9507	0.9544	0.9524	0.9526	0.9952
MobileNetV2	0.9392	0.9447	0.9417	0.9418	0.9942

It can be observed from the experimental results that DenseNet121 has outperformed the MobileNetV2 model, due to its dense connectivity pattern, which promotes better feature reuse and gradient flow, resulting in higher classification accuracy and improved generalization on unseen data. The class-wise performances for each of the models are being evaluated and presented in **Table 7**.

**Table 7:** Class-wise performance across local and global models.

Class	Model	Precision	Recall	F1-score	Accuracy
Late blight	Global DenseNet121	0.977 0.969	0.970 0.957	0.973 0.963	0.992 0.989
	MobileNetV2	0.966	0.938	0.952	0.985
*Septoria* leaf spot	Global DenseNet121	0.981 0.978	0.992 0.983	0.986 0.980	0.994 0.991
	MobileNetV2	0.975	0.978	0.976	0.989
Bacterial spot	Global DenseNet121	0.978 0.972	0.981 0.975	0.979 0.973	0.990 0.988
	MobileNetV2	0.966	0.964	0.965	0.986
Leaf mold	Global DenseNet121	0.981 0.975	0.983 0.970	0.982 0.972	0.991 0.987
	MobileNetV2	0.972	0.961	0.966	0.985
Early blight	Global DenseNet121	0.981 0.975	0.984 0.978	0.982 0.976	0.990 0.988
	MobileNetV2	0.972	0.957	0.964	0.986
Tomato mosaic virus	Global DenseNet121	0.986 0.986	0.990 0.976	0.988 0.981	0.995 0.992
	MobileNetV2	0.979	0.962	0.970	0.989
Tomato yellow leaf curl virus	Global DenseNet121	0.971 0.971	0.971 0.967	0.971 0.969	0.985 0.984
	MobileNetV2	0.971	0.963	0.967	0.983
Target spot	Global DenseNet121	0.978 0.978	0.987 0.987	0.982 0.983	0.992 0.992
	MobileNetV2	0.973	0.982	0.977	0.989
Spider mites	Global DenseNet121	0.976 0.971	0.967 0.953	0.971 0.962	0.987 0.982
	MobileNetV2	0.957	0.939	0.948	0.978
Powdery mildew	Global DenseNet121	0.937 0.937	0.967 0.967	0.952 0.952	0.981 0.981
	MobileNetV2	0.921	0.951	0.936	0.975
Healthy	Global DenseNet121	0.990 0.987	0.977 0.972	0.983 0.979	0.993 0.991
	MobileNetV2	0.979	0.961	0.970	0.988

Furthermore, the object detection head was evaluated to assess the robustness of the model. The object detection head was assessed with respect to mean average precision (mAP), average precision (AP), precision, recall, and F1-score. The observed values are presented in **Table 8**.

**Table 8:** Evaluation metrics for YOLOv8 and EfficientDet-D0 for weed detection.

Model	mAP@0.5	mAP@0.95	AP	Precision	Recall	F1-score
YOLOv8	0.956	0.732	0.823	0.942	0.928	0.935
EfficientDet-D0	0.978	0.786	0.865	0.969	0.954	0.961

Based on the quantitative evaluation presented in **Table 5**, EfficientDet-D0 demonstrates stronger overall performance compared to YOLOv8 across multiple detection metrics. YOLOv8 achieves higher values for mAP@0.5, mAP@0.95, average precision, precision, recall, and F1-score, indicating better localization accuracy. Although YOLOv8 shows stable convergence in its training curves, its final detection accuracy remains lower than that of EfficientDet-D0. These results confirm that EfficientDet-D0 provides a more robust and reliable detection framework for weed–crop identification in the evaluated setting. The observed per-class average precision is being assessed for all the classes across YOLOv8 and EfficientDet-D0 models as presented in **Table 9**.

**Table 9:** Per-class average precision of YOLOv8 and EfficientDet-D0 models.

Class	YOLOv8 AP@0.5	EffDet AP@0.5	YOLOv8 AP@0.95	EffDet AP@0.95
Late blight	0.948	0.972	0.726	0.777
*Septoria* leaf spot	0.952	0.975	0.728	0.781
Bacterial spot	0.951	0.974	0.725	0.780
Leaf mold	0.954	0.976	0.734	0.789
Early blight	0.957	0.979	0.733	0.788
Tomato mosaic virus	0.958	0.981	0.736	0.792
Tomato yellow leaf curl virus	0.960	0.982	0.735	0.791
Target spot	0.955	0.978	0.731	0.787
Spider mites	0.949	0.973	0.720	0.772
Powdery mildew	0.945	0.970	0.718	0.770
Healthy	0.956	0.979	0.732	0.786

**Figure 15:**
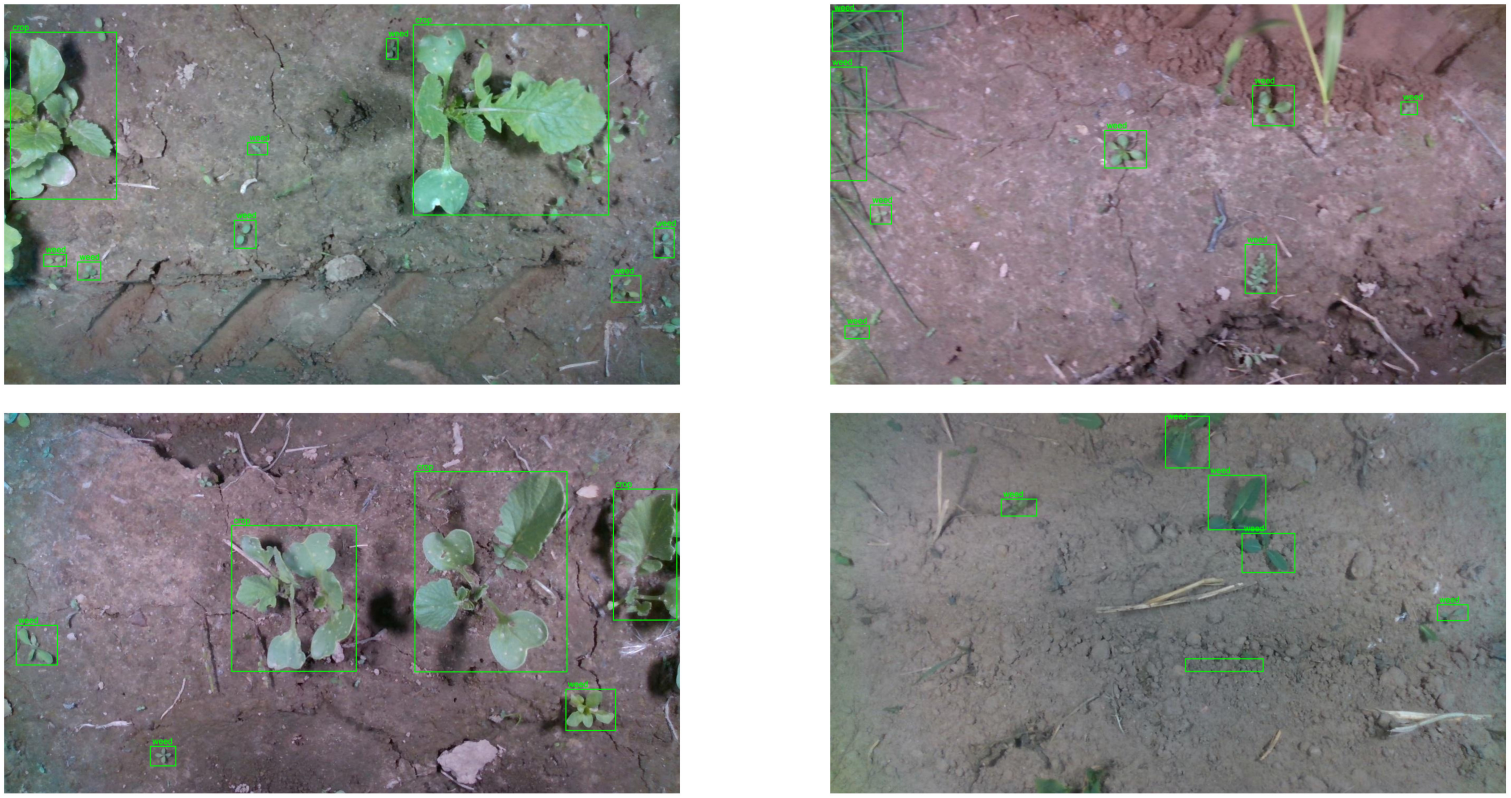
Results of the YOLOv8 model in detecting the crop and weed.

The global model’s detection head shares a backbone with the classification head and is trained in a multitask, federated setting, and this architectural and training setup can reduce localization performance relative to a locally trained YOLOv8 model. For transparency, the study has reported both mAP@0.5 and mAP@0.95 and emphasizes that interpretation should consider intersection over union (IoU) thresholds, per-class AP, and the effects of model aggregation in the federated scenario. The output screens of the YOLO V8 models are presented in **Figure 15**. The bounded box is created across the region of interest, and the label represents the associated object class.

**Figure 16:**
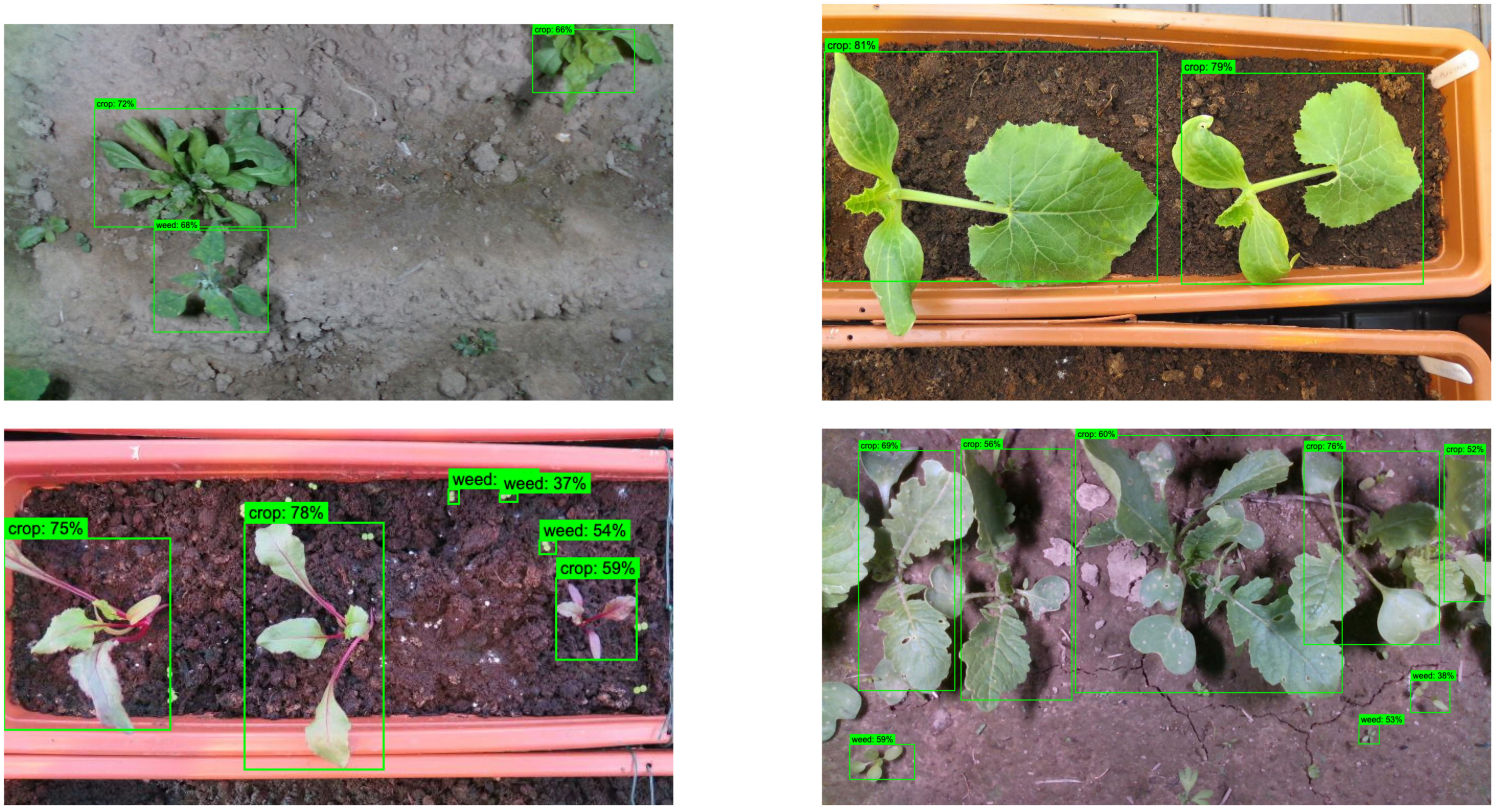
Results of the EfficientDet-D0 model in detecting the crop and weed.

Similarly, the outputs of the EfficientDet-D0 are presented in **Figure 16**. EfficientDet-D0 would also have the object enclosed inside the bounded box to highlight the region of interest. The object class is presented along with the confidence level of the prediction.

**Figure 17:**
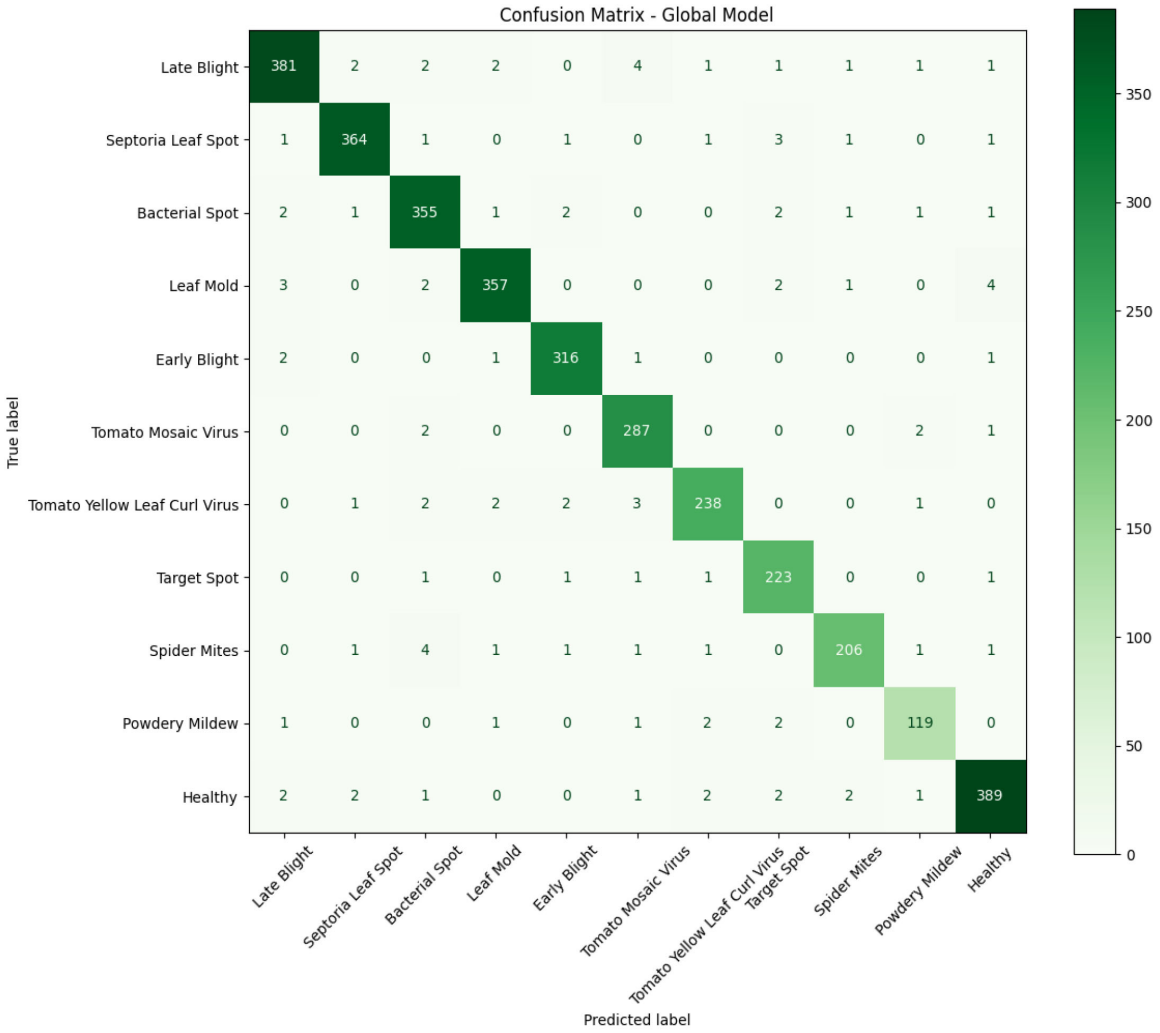
Confusion matrix of the global model associated with the tomato leaf disease dataset.

Furthermore, on updating the global model with the observed parameters from the local models, the model is further evaluated with respect to the same metrics. The obtained confusion matrix at the classification head of the global model is shown in **Figure 17**, and the observed values of the experimental outcome are presented in **Table 10**.

**Table 10:** Performance metrics of the global model for classification and object detection tasks.

Classification	Value	Object detection	Value
Accuracy	0.965	mAP@0.5	0.882
Precision	0.962	mAP@0.95	0.612
Recall	0.961	AP	0.874
F1-score	0.961	Precision	0.891
Specificity	0.981	Recall	0.862

The confusion matrices in **Figures 14**, **17** show that most predictions lie on the diagonal, confirming strong overall performance, but they also reveal systematic misclassifications concentrated among disease classes with highly similar visual symptoms. MobileNetV2 exhibits higher confusion between early blight, late blight, and target spot, reflecting its limited capacity to separate fine-grained lesion patterns. DenseNet121 reduces these errors but still shows occasional overlap between *Septoria* leaf spot and leaf mold, likely due to shared spot-like morphologies. In contrast, the global federated model further suppresses these ambiguities, with only minor residual errors in visually subtle classes such as powdery mildew and spider mites. These patterns indicate that federated aggregation enhances feature generalization across clients, leading to more robust discrimination of closely related disease categories.

It can be observed from the above table that the performance of the global model has outperformed the performance of the local models. This improvement highlights the strength of federated learning, where the aggregation of knowledge from multiple clients enables the model to generalize better and capture richer feature representations. As a result, the global model achieves higher accuracy, precision, and recall, ultimately leading to more reliable predictions for both leaf disease classification and weed detection tasks.

### 5.1 Future perspective model

The future perspective model of the proposed agentic AI-driven precision agriculture includes a mobile application that assists farmers and agro-agencies in providing timely care. The current perspective model would provide live updates on the farmland concerning the moisture content, temperature, and other climatic conditions. Such real-time information about the farmlands can assist farmers in delivering timely interventions and decision-making. The mobile application has four pages, which include a registration page, a login page, an input page for farm-related information, and a status page that delivers the current conditions of the agricultural farm.

**Figure 18:**
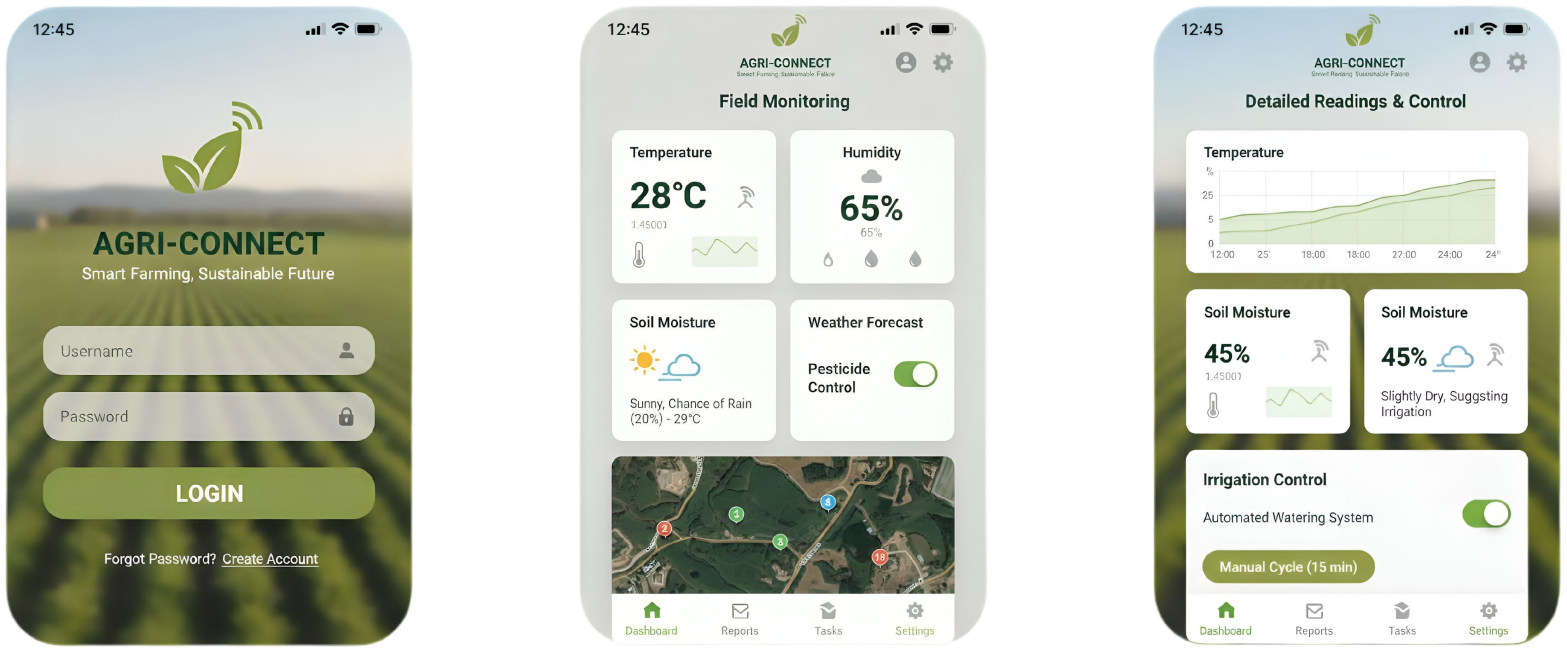
The GUI screens of the future perspective model.

A mobile application for an AAI-driven PA model can be integrated across multiple interfaces and libraries to ensure efficient real-time monitoring and a privacy-preserving FL environment. For on-device inference, TensorFlow Lite (TFLite) serves as the lightweight deployment framework, while NNAPI on Android and CoreML on iOS enable hardware-level acceleration through graphics processing unit (GPU) for faster processing of the input data and decision-making. To support decentralized training, TensorFlow Federated (TFF) can be employed, with TensorFlow Privacy ensuring differential privacy during model updates to protect farmers’ sensitive data. On the backend side of the application, the FastAPI provides a robust and lightweight interface to manage communication between devices, aggregate federated updates, and distribute improved models. Together, these technologies enable a secure, efficient, and scalable mobile application for real-time farm monitoring in precision agriculture. The sample graphical user interface (GUI) screens of the future perspective model are presented in **Figure 18**.

To advance the system from its current implementation toward a fully agentic form of intelligence, the future versions of the system will incorporate explicit agentic capabilities, which include the following things.

• Designing and developing the agents that are capable of doing autonomous goal formation, where agents dynamically define irrigation, nutrient, or pest-control goals based on learned field conditions rather than relying on static triggers.
• Integration of planning and self-directed task sequencing, allowing agents to design and carry out multistage operational plans.
• Incorporation of reflective learning mechanisms, allowing agents to reassess past actions and refine their behavior autonomously.

## 6 SWOT analysis

The current section of the manuscript covers the SWOT analysis on the role of AAI in PA, which outlines the practical implications and provides future research directions.

• *Strengths*: Agentic AI gives PA more power by letting it make its own smart decisions about all the tasks it is doing. This makes the best use of resources like water, fertilizers, and pesticides, which in turn boosts the quality and yield of the crops. AAI would also support real-time monitoring and predictive analytics, which would let farmers make timely and accurate changes that boost productivity. AAI helps make practices more sustainable by lowering their impact on the environment and encouraging the use of resources in a way that is good for the environment.
• *Weaknesses*: Despite its wider advantages and strengths in the PA domain, implementing AAI in agriculture faces some challenges. Small- and medium-sized farmers may have trouble getting started because of the high costs of the sensors, hardware, and AI infrastructure. Also, not all farmers have the technical knowledge they need to use the technology effectively. It can make it harder for agents to talk to each other and make decisions in real time, especially in remote areas that do not have good digital infrastructure. The latency introduced during synchronization rounds can slow real-time decision-making, especially in environments with unstable and low-throughput networks. Lastly, the concerns about data security and privacy can limit farmers’ willingness to share data required for effective system functioning.
• *Opportunities*: AAI offers broader opportunities in the PA domain, especially in the case of developing countries, that largely focus on sustainable and smart farming. The integration with smarter devices like IoT sensors, drones, remote sensing imaging equipment, and federated technologies can significantly enhance the traceability, scalability, and efficiency. Furthermore, context-aware and customizable AI systems that are tailored for robust monitoring of the crops, soil, and climate can boost farmer trust and support better localized farming strategies.
• *Threats*: Being the newer technology, AAI would also face resistance from farming communities, stemming from fear of loss of job opportunities in the associated fields, and unfamiliarity with the technology would slow down the transition process. High costs, adaptability challenges, and limited accessibility further hinder its adoption. Using energy on client devices may be a limitation in situations where there are many plans to expand, especially if model updates are done continuously. If the model size or the communication frequency is increased, the hardware requirements of the sensors deployed in the field or IoT devices may also be increased, thus causing problems of cost and sustainability.

## 7 Conclusion

The current study highlights the transformative potential of AAI in revolutionizing the PA. By leveraging intelligent agents with autonomous decision-making capabilities, the proposed framework demonstrates how farming practices can become more efficient, sustainable, and data-driven. The present work should be viewed as a foundational step toward agentic AI in precision agriculture rather than a complete agentic implementation. It illustrates the working mechanism of the AAI model, which highlights the decision-making process from the perception layer to the actuation layer. The case study outlines different types of AAI agents and their roles in sustainable farming, which also gives a practical roadmap for real-time implementation. The SWOT analysis that is presented in the current study would assist in better understanding the feasibility of using the technology. The findings from the case study provide concrete evidence supporting the potential of AAI as a promising pathway toward smarter farming ecosystems. The agentic framework, when combined with federated learning, enabled the global model to outperform all local models in both disease classification and weed detection, demonstrating improved generalization, reduced misclassification rates, and more robust feature extraction across heterogeneous data sources. These results illustrate how distributed agent-driven intelligence can enhance decision accuracy, support timely interventions, and operate effectively without requiring centralized data sharing. Taken together, the empirical improvements observed across classification, detection, and model aggregation directly justify the claim that AAI can enable more efficient, scalable, and data-driven precision agriculture systems.

The current model is just a fundamental outline of the feasibility of integrating AAI and FL technologies. However, the current study has not evaluated the dynamic and automated decision strategies of the AAI model. The multi-agent decision process of AAI technology is yet to be explored. The current case study is based on publicly available Kaggle datasets for training and evaluation, and it does not include real-world field validation. The current study can be extended by taking a real-time case study and analyzing various dynamic factors that would influence the adoptability of the technology in PA. Other factors, such as scalability, security, and robustness of the system, must be further analyzed to understand the performance of AAI models.

## Data Availability

Publicly available datasets were analyzed in this study. This data can be found here: The datasets used in the current study are openly accessible at https://www.kaggle.com/datasets/ashishmotwani/tomato and https://www.kaggle.com/datasets/vinayakshanawad/weedcrop-image-dataset/data.
